# Evidence for line width and carrier screening effects on excitonic valley relaxation in 2D semiconductors

**DOI:** 10.1038/s41467-018-04988-x

**Published:** 2018-07-03

**Authors:** Yuhei Miyauchi, Satoru Konabe, Feijiu Wang, Wenjin Zhang, Alexander Hwang, Yusuke Hasegawa, Lizhong Zhou, Shinichiro Mouri, Minglin Toh, Goki Eda, Kazunari Matsuda

**Affiliations:** 1 Institute of Advanced Energy, Kyoto University, Uji, Kyoto, 611-0011 Japan; 2 Graduate School of Science, Nagoya University, Chikusa, Nagoya, 464-8602 Japan; 3 Research Institute for Science and Technology, Tokyo University of Science, 6-3-1 Katsushika-ku, Tokyo, 125-8585 Japan; 4 Department of Electrical and Computer Engineering, Rice University, Houston, TX 77005 USA; 5 Department of Physics, Rice University, Houston, TX 77005 USA; 6 Department of Electrical and Electronic Engineering, Ritsumeikan University, Kusatsu, 525–8577 Japan; 7 Department of Physics, National University of Singapore, Singapore, 117551 Singapore; 8 Centre for Advanced 2D Materials and Graphene Research Centre, National University of Singapore, Singapore, 117546 Singapore; 9 Department of Chemistry, National University of Singapore, Singapore, 117543 Singapore; 10 Present Address: Department of Chemical Science and Technology, Hosei University, Koganei, Tokyo, 184-8584 Japan

## Abstract

Monolayers of transition metal dichalcogenides (TMDC) have recently emerged as excellent platforms for exploiting new physics and applications relying on electronic valley degrees of freedom in two-dimensional (2D) systems. Here, we demonstrate that Coulomb screening by 2D carriers plays a critical role in excitonic valley pseudospin relaxation processes in naturally carrier-doped WSe_2_ monolayers (1L-WSe_2_). The exciton valley relaxation times were examined using polarization- and time-resolved photoluminescence spectroscopy at temperatures ranging from 10 to 160 K. We show that the temperature-dependent exciton valley relaxation times in 1L-WSe_2_ under various exciton and carrier densities can be understood using a unified framework of intervalley exciton scattering via momentum-dependent long-range electron–hole exchange interactions screened by 2D carriers that depend on the carrier density and the exciton linewidth. Moreover, the developed framework was successfully applied to engineer the valley polarization of excitons in 1L-WSe_2_. These findings may facilitate the development of TMDC-based opto-valleytronic devices.

## Abstract (web summary)

Atomically thin transition metal dichalcogenides offer a platform to explore the valley degree of freedom originating from their electronic band structure. Here, the authors use polarization- and time-resolved spectroscopy to investigate the temperature-dependent valley pseudospin relaxation processes in WSe_2_ monolayers.

## Introduction

A “valley” is an electronic degree of freedom in momentum space, and its potential applications as information carriers in future electronics or optoelectronics devices are called valleytronics^1–4^. Monolayers of transition metal dichalcogenides (1L-TMDCs) *MX*_2_ (*M* = Mo, W; *X* = S, Se, etc.) have recently emerged as promising two-dimensional (2D) materials for developing valleytronics because they have hexagonal lattice structures similar to that of graphene but are semiconductors with finite direct band gaps in two inequivalent +*K* and −*K* valleys related by a time-reversal operation in the 2D hexagonal Brillouin zone^5,6^. Because of the reduced screening resulting from the atomically thin 2D structures of 1L-TMDCs, their excitons, which are mutually attracting electron–hole pairs that interact through Coulomb interactions, have extremely large binding energies and dominate their optical responses even at room temperature^7–10^. In addition, the strong spin–orbit interactions in these materials give rise to large spin splitting, reaching ~450 meV (~150 meV) for W*X*_2_ (Mo*X*_2_) in the valence band^11^. This large valence spin splitting and a lack of inversion symmetry in these materials lead to spin–valley coupling that enables exclusive access to the excitonic valley pseudospins (|+*K*〉 or |−*K*〉) with right- or left-circularly polarized photons^12–16^. These unique characteristics of 1L-TMDCs have provided unprecedented platforms for the study of valley-exciton physics in 2D systems, as well as offering opportunities for developing future optoelectronic devices using the excitonic valley degrees of freedom^4,12–15,17–33^.

One of the most intriguing questions in valley-exciton physics is in regard to the relaxation mechanism of excitonic valley pseudospins in 1L-TMDCs^4,12–15,17–23,28,30–32,34–48^. With regard to the theory on the subject, exciton (or hole) valley relaxations dominated by electron–hole (*e*–*h*) exchange interactions^14,19,20,32,38,39^, as well as phonon-assisted intervalley scattering mechanisms^13,17,37^, have been proposed and intensively studied. The importance of the Coulomb screening effect by naturally or intentionally doped carriers in 1L-TMDCs has also been examined theoretically^32^, although it has not yet been addressed experimentally. With regard to investigating the question experimentally, time- and polarization-resolved transient absorption^19,22^, reflection^35^, photoluminescence (PL)^28,41^, and Faraday^43^ and Kerr rotation (TRKR)^38,44,46,47,49–51^ techniques have been used to measure the depolarization (life) times of the spin or exciton (hole) valley states in 1L-WSe_2_^38,46,47,50,52^, 1L-WS_2_^22,49^, and 1L-MoS_2_^19,35,43,44,51^, and spin or valley relaxation times ranging from several to 10 ps^38,46,47^ to tens of nanoseconds^50^ have been reported at cryogenic temperatures. The considerable variations in the reported spin or valley relaxation times have been discussed as being possible consequences of either the differences between the physical quantities observed by each measurement technique^49^, the existence of dark exciton states^49^, the spin/valley polarization of resident holes^50,52^, ultrafast Coulomb-induced intervalley coupling^45^, the slow decay of localized states^47^, or the high excitation density typically needed for pump–probe-type experiments using femtosecond lasers (typically >~10^12^ cm^−2^ within the initial 100–200 fs)^28,46,49^. Because of all or some of these complications, a comprehensive and unifying explanation for temperature-dependent exciton valley relaxation times that is applicable for various exciton and carrier density conditions has yet to be provided.

In the present paper, we provide experimental evidence that the excitonic valley relaxation times and their temperature dependence in naturally carrier-doped 1L-TMDCs are dominated by momentum-dependent *e*–*h* exchange interactions screened by a 2D electron gas. We measured the valley polarization, linewidth, and time-dependent PL decay profile of excitons in 1L-WSe_2_ to examine the valley relaxation times of an exciton at temperatures ranging from 10 to 160 K in the linear response regime. We show that the low-temperature exciton valley relaxation times in 1L-WSe_2_ can be excellently reproduced using a framework of an intervalley exciton scattering mechanism via momentum-dependent long-range *e*–*h* exchange interactions screened by naturally doped 2D carriers. The temperature dependence of the Coulomb screening function deduced from the experimental data clearly demonstrates the 2D nature of the doped carriers; this is a unique manifestation of the true 2D structure of 1L-TMDCs. Moreover, we demonstrate that the developed framework can be used to predict the valley relaxation times and polarizations under various experimental conditions with various exciton linewidths, exciton lifetimes, carrier densities, and exciton densities. We also demonstrate that the valley polarization in 1L-WSe_2_ can be actually engineered via artificially modifying these parameters according to the theoretical prediction. Our findings therefore provide a unified framework through which the temperature-dependent exciton valley relaxation times and valley polarization in 1L-TMDCs can be understood and predicted; this framework may facilitate the development of TMDC-based opto-valleytronic devices.

## Results

### Measurements of exciton valley polarization

**Fig. 1:**
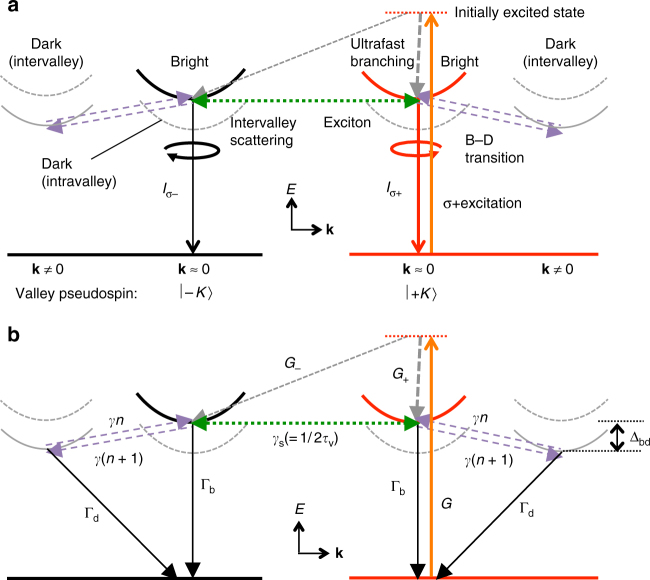
Excitation and relaxation processes of valley-polarized excitons. **a** Schematic of the processes in the |+*K*〉 valley-pseudospin-selective excitation followed by circularly polarized PL from the bright (**k** ≈ **0**) excitons with |+*K*〉 (red) or |−*K*〉 (black) valley pseudospins. Solid and dotted bands correspond to the singlet-like and triplet-like exciton bands, respectively. The horizontal axis corresponds to the exciton wave vector **k**. Exciton bands at **k** ≈ **0** are at the zone center in the excitonic Brillouin zone. Intervalley dark excitons have **k** ≠ **0**. Arrows indicate the major transition and scattering pathways assumed to be involved for the phenomena considered in this study. **b** Symbols denoting the rates for the exciton recombination and scattering processes considered in **a**. Parameter *γ* is a phonon scattering rate constant, and *n* is a phonon number available for the bright–dark (B–D) transitions with the energy difference of Δ_bd_. In the schematics of **a** and **b**, only the intervalley B–D scattering pathways are indicated for clarity. However, we do not exclude the possibility of the contribution of the intravalley B–D scattering (see Supplementary Note 1 for additional details)

Figure 1a shows a schematic of the excitation of optically allowed (bright) excitons with the center-of-mass wave vector **k** ≈ **0** in the excitonic Brillouin zone and the valley pseudospin |+*K*〉 (for which both electron and hole are in the +*K* valley in the electronic Brillouin zone), initial ultrafast decays partially branching into bright excitons with the valley pseudospin |+*K*〉 or |−*K*〉 (for which both electron and hole are in the −*K* valley in the electronic Brillouin zone), the intervalley scattering between the |+*K*〉 and |−*K*〉 states causing exciton valley relaxation, circularly polarized emission processes from the |+*K*〉 and |−*K*〉 bright excitons, and the electron spin-conserving scattering between the bright (**k** ≈ **0**) and dark (**k** ≠ **0**) exciton states in 1L-WSe_2_. The dark excitons with **k** ≠ **0** indicated by gray solid curves in Fig. 1 are optically forbidden because of the momentum mismatch with photons. Symbols *σ*_+_ and *σ*_–_ denote the circular polarizations, and $I_{\sigma + }$ and $I_{\sigma - }$ represent the corresponding PL intensities. Figure 1b shows the scattering processes and symbols for the relevant rates considered in this study. The valley relaxation time (*τ*_v_) of the bright exciton is related to its intervalley scattering rate (*γ*_s_) as *τ*_v_ = (2*γ*_s_)^−1^. *G* (=*G*_+_ + *G*_−_) is the generation rate of the bright excitons. Parameters *G*_+_ and *G*_−_ are the effective generation rate of the bright excitons with |+*K*〉 and |−*K*〉 pseudospins after the initial branching within the ultrafast timescale, respectively. We define *ρ*_0_ ≡(*G*_+_ − *G*_−_)/(*G*_+_ + *G*_−_) to express the valley polarization loss in the initial ultrafast non-thermal timescale. In the ideal case under *σ*_+_ excitation, such as the resonant excitation condition to the bright exciton state, *ρ*_0_ should be equal to 1 because of the optical selection rule^12–15^. However, *ρ*_0_ could be less than 1 in the event of an initial depolarization process due to defect-induced intervalley generation^35^, ultrafast valley relaxation of hot excitons during their intraband cooling processes^39^, and/or excitation to other spin-degenerate valleys^53^, potentially through phonon-assisted indirect absorption processes^37^. In Fig. 1, the exciton scattering processes between the bright and intervalley dark states by phonon-assisted spin-conserving electron scattering processes^54^ are tentatively indicated as a major bright–dark transition pathway. In this study, however, we do not attempt to distinguish the microscopic origin of the lower-lying dark states (intervalley spin-allowed or intravalley spin-forbidden) for the analysis under the condition that the hole’s valley is conserved. Recent experimental studies have revealed that the energy difference between the bright and dark states (Δ_bd_) in 1L-WSe_2_ is ~30–47 meV^54–57^. For the valley depolarization of holes (excitons), we consider the scattering between the singlet-like bright (**k** ≈ **0**) excitons with |+*K*〉 or |−*K*〉 valley pseudospins via long-range *e*–*h* exchange interactions^32,39^ to be the dominant valley depolarization mechanism. Because this mechanism is effective only between the two bright states with opposite valley pseudospins, the hole valley relaxation of dark excitons is neglected as a first approximation in this study (see Supplementary Note 1 for additional details).

**Fig. 2:**
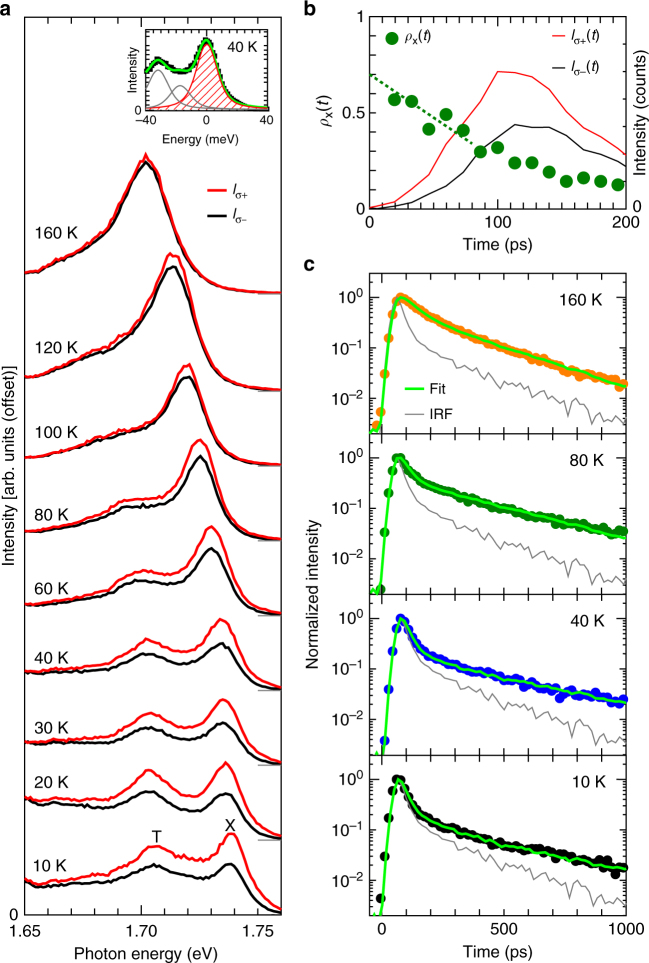
Polarization- and time-resolved PL measurements at various temperatures. **a** Polarization-resolved PL spectra of 1L-WSe_2_ on a quartz substrate under *σ*_+_ excitation at 10–160 K. The red and black curves correspond to the *σ*_+_ and *σ*_−_ PL intensities, *I*_*σ*__+_ and *I*_*σ*__−_, respectively. The inset shows an example of the spectral fitting for the *I*_*σ*__+_ spectrum at 40 K using Voigt functions. The shaded peak corresponds to the exciton PL. **b** *ρ*_x_ as a function of time (solid circles) calculated from time (*t*)-resolved PL signals of *I*_*σ*__+_(*t*) and *I*_*σ*__−_(*t*) at 45 K measured for 1L-WSe_2_ on a quartz substrate. **c** Time-resolved PL decay profiles of excitons measured at 160 K (orange), 80 K (green), 40 K (blue), and 10 K (black). Emission polarization was not resolved for these measurements. Light-green curves are fit to the data, and gray curves are the instrumental response function (IRF)

**Fig. 3:**
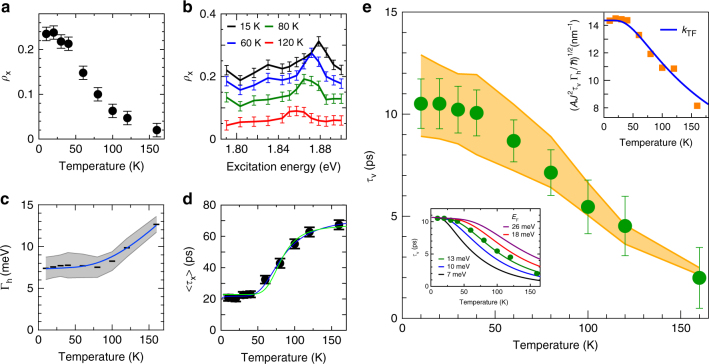
Temperature dependence of exciton valley relaxation times. **a** Valley polarization *ρ*_x_ as a function of temperature. **b** *ρ*_x_ at various temperatures as functions of excitation photon energy. The error bars in **a**, **b** correspond to the uncertainties of the polarization-resolved measurements. **c** Γ_h_ as a function of temperature. The gray-shaded region indicates the reasonable range of Γ_h_ deduced from the Voigt fitting procedure. Bars are the mean values in the reasonable range at each temperature. The blue curve is the empirical function obtained by the fit to the mean values. **d** 〈*τ*_x_〉 as a function of temperature. Solid curves are the fitting results obtained using Eq. (5) with Δ_bd_ of 30 meV (blue curve) and 47 meV (light-green curve). The error bars represent the uncertainties in the fitting procedure. **e** *τ*_v_ calculated using Eq. (1) and plotted as a function of temperature (solid circles). The error bars were estimated from the uncertainties in 〈*τ*_x_〉 and *ρ*_x_ used for calculating *τ*_v_. The orange-shaded region is the prediction band from Eq. (2) corresponding to the reasonable range of Γ_h_ in **c** with the fitting parameters of *J* = 0.83 eV and *E*_F_ = 13 meV (*n*_c_ ≈ 2.1 × 10^12^ cm^−2^). The upper-right inset is a plot of [*AJ*^2^*τ*_v_(*T*)Γ_h_(*T*)/*ħ*]^1/2^ as a function of temperature. The blue curve is the fitting result obtained by *k*_TF_(*T*, *E*_F_) with *E*_F_ = 13 meV. The lower-left inset shows the predicted temperature dependence of *τ*_v_ for various *E*_F_ (carrier density), as obtained using Eq. (3). For the Γ_h_, the empirical fit function plotted in **c** (blue curve) was used for clarity

Figure 2a shows the polarization-dependent PL spectra of 1L-WSe_2_ on a quartz substrate under circularly polarized *σ*_+_ excitation conditions at 10–160 K. At low temperatures, an exciton (A-exciton) peak (X, at 1.740 eV)^34,58^ and a charged exciton (trion) peak (T, at 1.705 eV)^34^ were observed. The emergence of the trion peaks and their peak positions indicate that 1L-WSe_2_ was in a naturally electron-doped condition^34,59^. The difference between the spectral integrated intensities of *σ*_+_ (*I*_*σ*__+_) and *σ*_–_ (*I*_*σ*__−_) exciton PL (valley polarization) was clearly observed at low temperatures. Under the excitation by *σ*_+_ photons, the valley polarization, *ρ*_x_, is calculated as *ρ*_x_ = (*I*_*σ*__+_ − *I*_*σ*_−)/(*I*_*σ*__+_ + *I*_*σ*__−_). As shown in Fig. 3a, *ρ*_x_ values were nearly constant under the low-temperature conditions (*T* < ~40 K) and gradually decreased with increasing temperature for *T* > ~60 K. Similar temperature-dependent behaviors of *ρ*_x_ have been reported for 1L-MoS_2_^13^ and 1L-WSe_2_ (for *T* ≥ 70 K)^31^.

The excitation photon energy dependences of *ρ*_x_ at various temperatures were also examined; the results are shown in Fig. 3b. Relatively high values of *ρ*_x_ were observed at the near-resonant excitation conditions with the 2*s* excited exciton state^8,9^ lying at an energy ~0.14 eV higher than the 1*s* excitons at 1.74 eV, whereas variations in *ρ*_x_ were small under the nonresonant conditions (corresponding to excitation photon energies of ~1.79–1.84 eV and 1.89–1.90 eV) within the measured range from 1.79 to 1.90 eV. Because we employed nonresonant excitation conditions (the excitation photon energy was 1.893 eV) for evaluating the temperature dependence of *ρ*_x_ shown in Fig. 3a, we ruled out the possibility that the observed temperature dependence in *ρ*_x_ was determined by the temperature-dependent exciton energy shift^60^, which had previously been discussed as being the case in 1L-MoS_2_^14,40^. The excitation photon energy dependence of *ρ*_x_ at low temperature is discussed in greater detail in Supplementary Note 2 and Supplementary Figure 1.

To deduce the valley polarization loss in the initial ultrafast non-thermal timescale (the value of *ρ*_0_) for the following analysis, we also measured polarization-resolved PL decay profiles of the excitons in 1L-WSe_2_ on a quartz substrate. Figure 2b shows the polarization-resolved PL decay profiles [*I*_*σ*__+_ (red curve) and *I*_*σ*_− (black curve)] and the time-resolved valley polarization, *ρ*_x_(*t*), (green circles) measured at 45 K under the *σ*_+_ excitation condition. By extrapolating *ρ*_x_(*t*) to the onset time (*t* ≈ 0 ps) of the PL decay signals (dotted line), the lower limit of the maximum valley polarization of *ρ*_0_ ≡ *ρ*_x_(0) ≈ 0.7 ± 0.1 is deduced. Since the similar value was also deduced at 120 K (see Supplementary Note 3 and Supplementary Figure 2 for additional details), temperature-dependent variation of the *ρ*_0_ parameter is considered to be small in the observed temperature range, and will be neglected in the following analyses (See also Supplementary Note 4 and Supplementary Figure 3 for the effects of the uncertainty in the *ρ*_0_ on the following analysis).

### Temperature variations in exciton linewidth

Information about the temperature-dependent variations of the exciton homogeneous linewidths Γ_h_ were deduced from the PL spectral analysis. We evaluated Γ_h_ using a fitting procedure with Voigt functions (see Methods, Supplementary Note 5, and Supplementary Figure 4 for the detailed procedure). The inset in Fig. 2a shows an example of the peak deconvolution employed for the spectrum collected at 40 K. The reasonable uncertainty range of Γ_h_ estimated from the fit analysis (gray-shaded region) is plotted as a function of temperature in Fig. 3c. The temperature dependence of Γ_h_ was phenomenologically modeled as Γ_h_ = Γ_0_ + Γ_1_*T* + Γ_2_[exp(*E*_ph_/*k*_B_*T*) − 1]^−1^, where *k*_B_ is the Boltzmann constant, Γ_0_ is the temperature-independent linewidth, Γ_1_ and Γ_2_ are the fitting parameters, and *E*_ph_ = 31 meV is assumed to be a typical optical phonon energy in 1L-WSe_2_^61^. For convenience, we fit the mean values of Γ_h_ within a reasonable range at each temperature and obtained a best fit with Γ_0_ = 7.3 meV, Γ_1_ = 0.004 meV/K, and Γ_2_ = 39 meV. The fitted curve is plotted in Fig. 3c. The observed temperature dependence is consistent with trends previously reported for 1L-WSe_2_^60,62–64^.

### Valley relaxation time of the bright excitons

We now consider the relationship between the valley polarization *ρ*_x_ and the pure valley relaxation time *τ*_v_ of the bright excitons. The time-integrated valley polarization of the excitons can be expressed by the following phenomenological equation^14,15,40,65^ (see Supplementary Note 1 for additional details):

1 $$\rho _{\mathrm{x}} = \frac{{\rho _0}}{{1 + \left\langle {\tau _{\mathrm{x}}} \right\rangle /\tau _{\mathrm{v}}}}.$$

We set *ρ*_0_ = 0.7 for 1L-WSe_2_ in the following analyses according to the results discussed in the previous section. Parameter 〈*τ*_x_〉 in Eq. (1) corresponds to an exciton population lifetime in the simplest case for which only the bright exciton levels are involved^14^. For the multi-exciton-level system we consider in this study (Fig. 1), 〈*τ*_x_〉 is defined as an effective lifetime of the bright excitons that corresponds to the total (integrated) time in which a single exciton remains in its bright state before it recombines radiatively or nonradiatively (see also Supplementary Note 1). Equation (1) suggests that *τ*_v_, which is defined as the pure valley relaxation time of the bright excitons, can be evaluated if *ρ*_0_ and 〈*τ*_x_〉 are given. This procedure enables the evaluation of the pure valley relaxation time of an exciton in its bright state, which can be directly compared against the theoretical results. Notably, Eq. (1) is valid under the condition that the valley relaxation of holes composing excitons is dominated by the intervalley scattering of the bright excitons regardless of the microscopic origin of the scattering.

We evaluated 〈*τ*_x_〉 from the time-resolved PL decay profile of the bright excitons, *I*(*t*), as $\left\langle {\tau _{\mathrm{x}}} \right\rangle = {\int_{0}}^\infty {I\left( t \right)/I\left( 0 \right){\mathrm{d}}t}$, where *I*(*t*) is obtained by a fitting of the experimental data using a model PL decay function. Figure 2c shows representative PL decay profiles at 10, 40, 80, and 160 K in semi-log plots (see also Supplementary Note 6 and Supplementary Figure 5 for the data in linear plots). The data at each temperature were fitted as a convolution of the instrumental response function (IRF) and a model double exponential function (light-green curves), *I*(*t*) = *C*_1_exp(−*t*/*τ*_1_) + *C*_2_exp(−*t*/*τ*_2_), and 〈*τ*_x_〉 = (*C*_1_*τ*_1_ + *C*_2_*τ*_2_)/(*C*_1_ + *C*_2_) was obtained by the fitting procedure. Figure 3d plots the obtained 〈*τ*_x_〉 as a function of temperature (See Supplementary Note 7 and Supplementary Figure 6 for the consistency between the temperature dependences of the 〈*τ*_x_〉 and the exciton PL intensity). The solid curves represent the fit obtained using a model for Eq. (5) described in the Methods section (and in Supplementary Note 1), where the exciton scattering pathways shown in Fig. 1b were considered. We found that the temperature dependence of 〈*τ*_x_〉 can be well fitted using Eq. (5) with the bright–dark energy splitting^54–57^ Δ_bd_ in the range 30–47 meV, supporting the validity of the model of the multi-exciton-level system shown in Fig. 1 (see Supplementary Note 1 for more detailed discussion on the results of the fitting and limitation of the model).

### Valley relaxation mechanism of excitons

We then deduced the *τ*_v_ from the relation of Eq. (1), namely, *τ*_v_^−1^ = 〈*τ*_x_〉^−1^ (*ρ*_0_/*ρ*_x_ − 1), using the experimentally deduced values of *ρ*_0_, *ρ*_x_, and 〈*τ*_x_〉 at each temperature. Figure 3e shows the obtained valley relaxation times at various temperatures (green circles). The *τ*_v_ values under the low-temperature conditions (<~40 K) were on the order of 10 ps and exhibited plateau-like behavior. Here, we discuss the origin of the exciton valley relaxation in greater detail on the basis of the temperature dependence of the bright exciton valley pseudospin relaxation times, *τ*_v_. According to previous theoretical predictions^32,39^, we consider long-range *e*–*h* exchange interactions (originally known as the Maialle–Silva–Sham (MSS) mechanism for exciton spin-depolarization in semiconductors^66^) screened by 2D electrons naturally doped in 1L-WSe_2_ to be the dominant relaxation mechanism of the valley pseudospin of the bright excitons^32^. In this theoretical framework, the inverse exciton valley relaxation time is given as, *τ*_v_^−1^ = 〈 Ω_**k**_^2^*τ*_h_〉, where Ω_**k**_ is the momentum-dependent Larmor frequency under an effective magnetic field and *τ*_h_ is the momentum relaxation time, which is inversely proportional to Γ_h_ (See also Supplementary Note 8). Under low-temperature conditions (Γ_h_ > ~*k*_B_*T*) with screening by doped carriers, *τ*_v_ for bright excitons can be approximately expressed as^32^

2 $$\frac{1}{{\tau _{\mathrm{v}}}} = \frac{{AJ^2\Gamma _{\mathrm{h}}}}{\hbar }\frac{1}{{k_{{\mathrm{TF}}}^2}},$$

where $A = 9a^2M^2/4\pi ^2\hbar ^4.$ is a material-dependent constant in which *a* is the lattice constant, *M* is the exciton mass, $\hbar$ is Planck’s constant divided by 2*π*, *J* is the strength of the exchange interaction, and *k*_TF_ is the Thomas–Fermi wave vector; *k*_TF_ has a dimensionality-dependent function form and, for a 2D electron gas, *k*_TF_(*T*, *E*_F_) = *k*_TF0_[1−exp(−*E*_F_/*k*_B_*T*)]^32^. Parameter *E*_F_ is the Fermi energy measured from the bottom of the conduction band, and it is related to the carrier density, *n*_c_, by *n*_c_ = *g*_s_*g*_v_*m*_*e*_*E*_F_/(2π*ħ*^2^), where *g*_s_ and *g*_v_ are the spin and valley degeneracies, respectively, and *m*_e_ is the electron mass. Parameter *k*_TF0_, given by $k_{{\mathrm{TF0}}} = g_{\mathrm{s}}g_{\mathrm{v}}m_{\mathrm{e}}e^2/\left( {4\pi \varepsilon \hbar ^2} \right)$, is a zero-temperature Thomas–Fermi wave vector. In the following analyses, *a* = 0.334 nm, *m*_e_ = 0.38*m*_0_^67^, and *M* = 2*m*_e_ were used for 1L-WSe_2_. Equation (2) is valid when *k*_h_ ≪ *k*_TF_, where $k_{\mathrm{h}} = \sqrt {2M\Gamma _{\mathrm{h}}/\hbar ^2}$ is a maximum wave vector of excitons determined by the collisional broadening; this condition is safely met for a carrier density of *n*_c_ > ~5 × 10^10^ cm^−2^ for Γ_h_ < ~35 meV, which is normally fulfilled in naturally carrier-doped 1L-TMDCs.

The orange-shaded region in Fig. 3e is the prediction band for valley relaxation times reproduced using Eq. (2) and the experimentally deduced values of Γ_h_ within a reasonable uncertainty range (gray-shaded region in Fig. 3c) with fitting parameters of *E*_F_ (∝*n*_c_) and *J* for the screening function. The vertical width of the prediction band mainly originates from the uncertainty in Γ_h_. The temperature dependence of *τ*_v_ deduced using Eq. (1) (solid circles) could be excellently reproduced using the theoretical model of Eq. (2) with the experimental Γ_h_, given the fitting parameters *E*_F_ = 13 meV (*n*_c_ ≈ 2.1 × 10^12^ cm^−2^) and *J* = 0.83 eV. The implied *E*_F_ and the carrier density on the order of 10^12^ cm^−2^ is consistent with the observed trion PL intensity (see also Supplementary Note 9) and previous results for as-exfoliated 1L-TMDCs^68^. Within the **k**·**p** approximation, *J* can be roughly estimated as *J* ≈ 8*π*^2^*aE*_b_*t*^2^/3*a*_B_*E*_g_^2^ ^32,69^, where *E*_b_ is the exciton-binding energy, *t* is the hopping energy, *E*_g_ is the band gap, and *a*_B_ is the exciton Bohr radius. Using *E*_b_ = 0.37 eV^8^, *t* = 1.19 eV^12^, *E*_g_ = 2.11 eV, and 1 ≤ *a*_B_ ≤ 2 nm for 1L-WSe_2_, approximately 0.5 ≤ *J* *≤* 1 eV is deduced. Thus, the fitting result of *J* ≈ 0.83 eV is consistent with the theoretical prediction of the exchange interaction strength.

The lower-left inset of Fig. 3e shows a plot of the *E*_F_ dependence of *τ*_v_ calculated using Eq. (2) and the empirical function for the mean values of Γ_h_. The low-temperature values of *τ*_v_ are clearly observed to be independent of *E*_F_, and the low-temperature plateau is extended for larger *E*_F_ values (higher carrier density). For the intermediate temperatures (~40 K < *T* < ~160 K), the valley relaxation times will be longer when the carrier density is greater. For further high-temperature conditions, Eq. (2) may no longer be applicable. At high temperatures, valley depolarization mechanisms other than the screened *e*–*h* exchange interactions, such as the intervalley scattering through the thermal activation of holes to the spin-degenerate Γ valley^22^, may also coexist, which would lead to Arrhenius-type temperature dependence with an activation energy corresponding to the energy difference of the |*K*_e_*K*_h_〉 and |*K*_e_Γ_h_〉 excitons with electrons in the ±*K* valley and holes at the ±*K* or Γ valley. The activation energy has been estimated to be on the order of 0.14 eV for 1L-WS_2_^22^; assuming a similar order for 1L-WSe_2_, this scattering pathway becomes gradually important for *T* > ~ 200 K.

To show the manifestation of the 2D screening in *τ*_v_(*T*) more clearly, we plotted $\sqrt {AJ^2\tau _{\mathrm{v}}\left( T \right)\Gamma _{\mathrm{h}}\left( T \right)/\hbar }$ in the right-top inset of Fig. 3e, which corresponds to *k*_TF_(*T*, *E*_F_) according to Eq. (2). The mean values of the estimated linewidth in the uncertainty range shown in Fig. 3c were used as Γ_h_(*T*) at each temperature. The function form of the characteristic 2D Thomas–Fermi wave vector, *k*_TF_(*T*, *E*_F_), excellently reproduces the temperature dependence originating from the term $\sqrt {\tau _{\mathrm{v}}\left( T \right)\Gamma _{\mathrm{h}}\left( T \right)}$. This plot and Eq. (2) also suggest that the dimensionless quantity $k_{{\mathrm{TF}}0}^2/AJ^2 = \tau _{\mathrm{v}}\left( 0 \right)\Gamma _{\mathrm{h}}\left( 0 \right)/\hbar$ is a constant that is dependent only on the material and is directly accessible by experiment. From our current experimental results, we obtained *τ*_v_(0)Γ_h_(0)/*ħ* ≈ 118 for 1L-WSe_2_. Although the aforementioned considerations suggest that the presented model could capture important physics in the exciton valley relaxation phenomenon in doped 1L-WSe_2_, nevertheless, we note that our theoretical treatment within the static approximation neglects the doping-dependent changes of the exciton-binding energy and the quasiparticle band gap^68^ dominated by the dynamical screening effect on the direct Coulomb interactions^70^, and any possible dynamical effect on the screening of the *e*–*h* exchange interactions; these potential shortcomings may change the formula of the valley relaxation time both in a qualitative and quantitative manner (See Supplementary Note 10 for more detailed discussion). Thus, it is still desired to develop a more rigorous theoretical approach for in-depth understanding of the underlying physics on the valley relaxation phenomena in the carrier-doped 2D semiconductors.

### Valley polarization engineering

On the basis of the aforementioned results, a semi-empirical formula for the temperature-, Fermi energy- (carrier density-), and linewidth-dependent *τ*_v_ can be expressed as:

3 $$\tau _{\mathrm{v}}\left( {T,E_{\mathrm{F}},\Gamma _{\mathrm{h}}} \right) = \frac{{C\hbar \left[ {1 - \exp \left( { - E_{\mathrm{F}}/k_{\mathrm{B}}T} \right)} \right]^2}}{{\Gamma _{\mathrm{h}}}},$$

where *C* is a material-dependent dimensionless parameter *C* ≡ *k*_TF0_^2^/A*J*^2^ = *τ*_v_(0)Γ_h_(0)/*ћ* (see Supplementary Note 10 for further discussion on the material dependence of *C*). From the experimental values of *τ*_v_ and Γ_h_ at the low-temperature condition, *C* ≈ 118 for the 1L-WSe_2_ was deduced in this study. Then, upon substitution of Eq. (3) into Eq. (1), the steady-state exciton valley polarization is predicted as:

4 $$\rho _{\mathrm{x}} = \frac{{\rho _0}}{{1 + \left\langle {\tau _{\mathrm{x}}} \right\rangle \Gamma _{\mathrm{h}}/C\hbar \left[ {1 - \exp \left( { - E_{\mathrm{F}}/k_{\mathrm{B}}T} \right)} \right]^2}}.$$

Equation (4) suggests a condition in which the exciton valley polarization becomes high; the narrower linewidth Γ_h_, the larger *E*_F_ (carrier density), and the shorter 〈*τ*_x_〉 will be the keys to achieving a longer *τ*_v_ and a higher *ρ*_x_. We first examined the expected correlation between the *ρ*_x_ and the Γ_h_ by changing the excitation power density at 10 K using Eq. (4) at the low-temperature limit *ρ*_x_ ≈ *ρ*_0_(1+〈*τ*_x_〉Γ_h_/*Cћ*)^−1^. The expected inverse correlation of *ρ*_x_ and Γ_h_ was actually observed and could be well reproduced using this simple relation, as shown in Supplementary Note 11 and Supplementary Figure 7. Moreover, we could also successfully apply the above relation for reproducing the excitation photon energy dependence of the valley polarization observed at 15 K from the 〈*τ*_x_〉 and Γ_h_ observed at each excitation photon energy (Supplementary Note 2 and Supplementary Figure 1). The wide applicability of the aforementioned relation under low-temperature conditions thus strongly supports the validity of the presented mechanism.

**Fig. 4:**
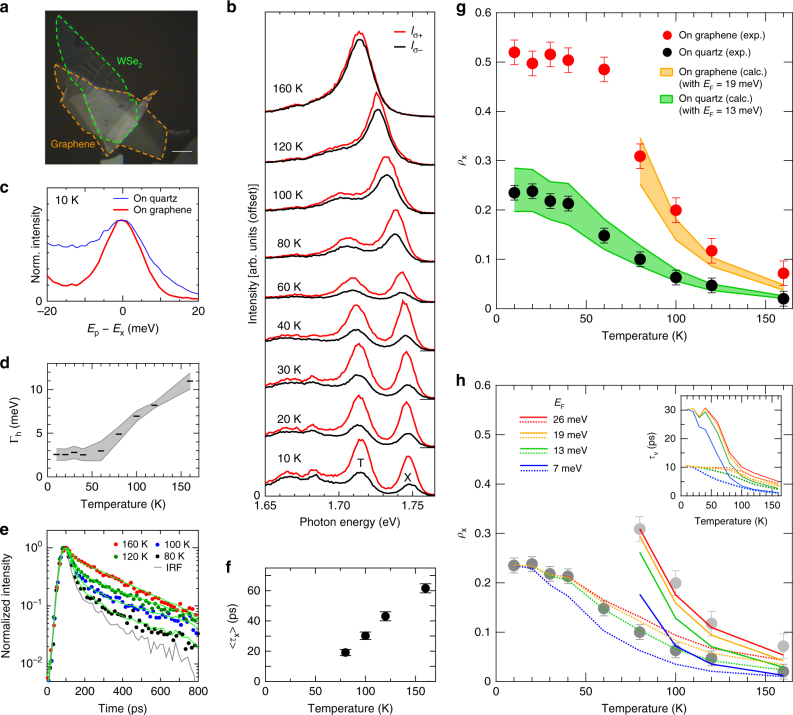
Valley polarization engineering. **a** Optical microscope image of 1L-WSe_2_ stacked on a multilayer graphene flake (on-graphene) on a quartz substrate. The scale bar indicates 10 μm. **b** Polarization-resolved PL spectra of the on-graphene 1L-WSe_2_ under *σ*_+_ excitation at 10–160 K. The red and black curves correspond to the *σ*_+_ and *σ*− PL intensities, *I*_*σ*__+_ and *I*_*σ*__−_, respectively. **c** Comparison of the exciton PL line shapes at 10 K for the on-graphene and the on-quartz 1L-WSe_2_ samples. **d** Γ_h_ as a function of temperature for the on-graphene 1L-WSe_2_. Gray-shaded region indicates the uncertainty range of Γ_h_ deduced from the Voigt fitting procedure. Bars are the mean values in the uncertainty range at each temperature. **e** Time-resolved PL decay profiles of excitons in the on-graphene 1L-WSe_2_ measured at 160 K (red), 120 K (green), 100 K (blue), and 80 K (black), respectively. **f** 〈*τ*_x_〉 in the on-graphene 1L-WSe_2_ plotted as a function of temperature. The error bars represent the uncertainties in the fitting procedure. **g** *ρ*_x_ as a function of temperature for the on-graphene 1L-WSe_2_ (red circles) compared with those of on-quartz (black circles, same data are shown in Fig. 3a) 1L-WSe_2_. The orange-shaded region is the prediction band of *ρ*_x_ for the on-graphene 1L-WSe_2_ calculated using Eq. (1); *ρ*_0_ = 0.7, 〈*τ*_x_〉 plotted in **f**, and *τ*_v_ calculated using Eq. (3) (with the Γ_h_ in the uncertainty range shown in **d** and *E*_F_ = 19 meV) were used as input parameters for Eq. (1). The green-shaded region is a prediction band for the on-quartz 1L-WSe_2_ with *E*_F_ = 13 meV. The error bars correspond to the uncertainties of the polarization-resolved measurements. **h** *ρ*_x_ predicted for various *E*_F_ as functions of temperature. The inset shows *τ*_v_ calculated using Eq. (3) with the mean values of Γ_h_ shown in **d** and various *E*_F_ values. Solid and dotted curves are for on-graphene and on-quartz samples, respectively

To further verify Eq. (4) at the finite-temperature conditions and achieve realistic engineering on the excitonic valley polarization, we fabricated 1L-WSe_2_ stacked on a multilayer graphene flake, as shown in Fig. 4a (see Methods). For this type of sample, we expect that the exciton linewidth could be narrow because of the reduced scattering on the atomically flat surface of multilayer graphene^71^ and because of the shorter 〈*τ*_x_〉 resulting from charge or energy transfer to the graphene. Modulating the carrier density in 1L-WSe_2_ may be possible because of the chemical potential difference between the graphene and 1L-WSe_2_.

Figure 4b shows the temperature dependence of the exciton valley polarization in 1L-WSe_2_ stacked on the multilayer graphene substrate (on-graphene sample). For this sample, we observed a much higher exciton valley polarization *ρ*_x_ compared with those for the 1L-WSe_2_ on the quartz substrate (on-quartz sample), as compared in Fig. 4g. As expected, exciton linewidths much narrower than those on the quartz substrate were observed (Fig. 4c, d). In addition, the trion peak intensity was higher than the exciton’s peak intensity, which suggests that the carrier density in this sample was higher than that in the sample on the quartz substrate. The trion/exciton intensity ratio for the on-graphene sample shown in Fig. 4b is ~1.5 times greater than that for the on-quartz sample shown in Fig. 2. We also observed the PL decay profiles (Fig. 4e). We could obtain the 〈*τ*_x_〉 for temperatures only more than 80 K as shown in Fig. 4e, f because of the detection limit. The orange-shaded region in Fig. 4g is the prediction band for the *ρ*_x_ reproduced using the 〈*τ*_x_〉 and Γ_h_ values shown in Fig. 4f, d with *E*_F_ = 19 meV (*n*_c_ ≈ 3.0 × 10^12^ cm^−2^) as inputs to Eq. (4). For comparison, the prediction band for the on-quartz sample (green-shaded region) calculated with the Γ_h_ shown in Fig. 3c and *E*_F_ = 13 meV (*n*_c_ ≈ 2.1 × 10^12^ cm^−2^) is also shown.

The valley polarization of the on-quartz sample and the increased valley polarization for the on-graphene sample could be consistently reproduced using Eq. (4). Importantly, the persistent valley polarization for *T* > ~100 K in the on-graphene 1L-WSe_2_ cannot be explained by simply considering that the 〈*τ*_x_〉 in the on-graphene sample is shorter than that in the on-quartz sample. For instance, at 100 K, the *ρ*_x_ for the on-quartz sample was only ~0.06 and 〈*τ*_x_〉 was ~55 ps. The *τ*_v_ was then deduced to be ~5.5 ps, as shown in Fig. 3e. If 〈*τ*_x_〉 becomes shorter, such as 〈*τ*_x_〉 ≈ 30 ps, as observed in the on-graphene sample, and the other parameters in Eq. (1) are kept unchanged, then *ρ*_x_ is expected to increase only slightly to ~0.1. This prediction contradicts the much higher *ρ*_x_ ≈ 0.2 for the on-graphene 1L-WSe_2_ at 100 K; thus, the narrower Γ_h_ and higher carrier density also contribute to the high-valley polarization, as predicted from Eq. (4). Figure 4h shows the *E*_F_ dependence of the *ρ*_x_ predicted by Eq. (4). For clarity, we used the mean values of the Γ_h_ in the uncertainty ranges shown in Fig. 3c (on-quartz) and Fig. 4d (on-graphene). The high values of *ρ*_x_ for the on-graphene sample clearly cannot be reproduced with the same *E*_F_ (carrier density) as that for the on-quartz sample. The inset of Fig. 4h shows the *τ*_v_ for various *E*_F_ calculated using Eq. (3) and the mean values of the Γ_h_ for the corresponding samples. The plateau region of *τ*_v_ is predicted to be extended as the *E*_F_ increased, consistent with the persistent *ρ*_x_ for the on-graphene sample at temperatures as high as ~60 K (Fig. 4g). These results confirm that the presented framework provides clear guidelines for engineering exciton valley polarization in 1L-WSe_2_. We note that there remains a possibility that the *ρ*_0_ and *C* also vary depending on the carrier density and/or the substrate’s dielectric constant. Thus, the prediction of Eq. (4) may be further modified when taking these effects into account (See Supplementary Note 10 for more detailed discussion).

## Discussion

Finally, we discuss whether our results can be extended so as to predict the exciton valley relaxation times in 1L-WSe_2_ under high-excitation-density conditions employed in ultrafast spectroscopic studies using femtosecond lasers^38,46^. The temperature dependence of *τ*_v_ deduced in this study, as shown in Fig. 3, is qualitatively similar to the temperature dependence of previous results obtained by TRKR measurements^38,46^; however, the absolute values we obtained are a few times greater than those previously reported. We address this discrepancy through the exciton density dependence of the linewidth. A previous study on the intrinsic excitonic linewidth in 1L-WSe_2_^62^ reported that Γ_h_ strongly depends on the excitation density, *N*_x_, as Γ_h_ = Γ_s_ + Γ_col_*N*_x_, under intense excitation conditions using femtosecond pulsed lasers, where Γ_s_ is a single exciton linewidth and Γ_col_ ≈ 5.4 × 10^12^ meV cm^−2^ is the constant for the density-dependent broadening due to exciton–exciton collisions^62^. Thus, we assume that the density- and temperature-dependent linewidths are expressed as Γ_h_(*T*, *N*_x_) = Γ_s_(*T*) + Γ_col_*N*_x_, where Γ_s_(*T*) is the linewidth observed under the weak excitation conditions and Γ_col_ is a temperature-independent proportionality constant.

**Fig. 5:**
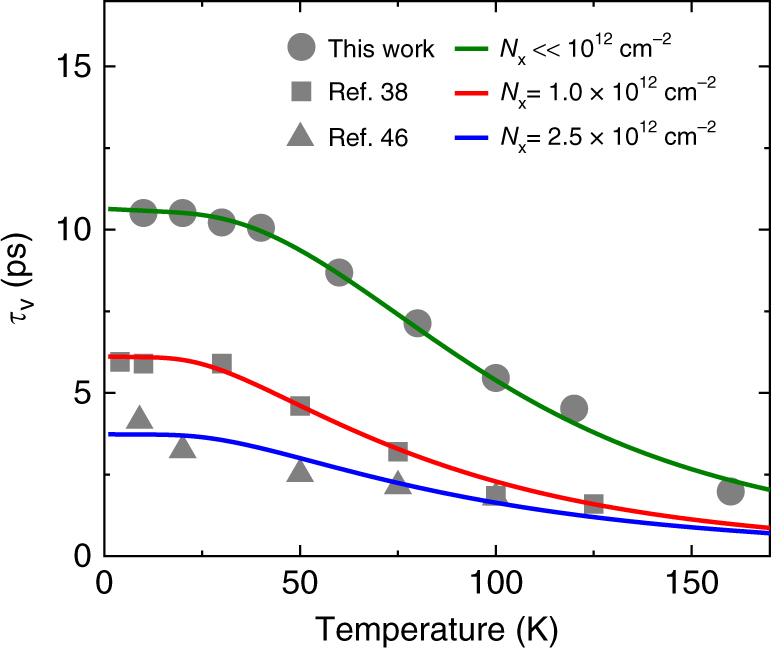
Temperature dependence of exciton valley relaxation times predicted for various exciton density conditions. Temperature-dependent exciton valley relaxation times obtained for 1L-WSe_2_ on the quartz substrate in this study (circles), and those previously reported in ref. ^38^ (squares) and ref. ^46^ (triangles), both of which were measured using the TRKR technique. The green, red, and blue curves were reproduced using Eq. (3) and the density-dependent Γ_h_ (*T*, *N*_x_) with *N*_x_ ≪ 10^12^ cm^−2^ (Γ_h_(0, *N*_x_) = 7.3 meV, green curve), *N*_x_ = 1.0 × 10^12^ cm^−2^ (Γ_h_(0, *N*_x_) = 12.7 meV), and 2.5 × 10^12^ cm^−2^ (Γ_h_(0, *N*_x_) = 20.8 meV, blue curve), respectively. The *E*_F_ parameters were set as 13 meV (*n*_c_ ≈ 2.1 × 10^12^ cm^−2^) for the green curve, 9 meV (*n*_c_ ≈ 1.4 × 10^12^ cm^−2^) for the red curve, and 10 meV (*n*_c_ ≈ 1.6 × 10^12^ cm^−2^) for the blue curve, respectively, to reproduce the experimental temperature dependence

Figure 5 shows the *τ*_v_ for various exciton densities, *N*_x_, in 1L-WSe_2_ predicted using the exciton-density-dependent Γ_h_(*T*, *N*_x_) as inputs for Eq. (3). For the temperature-dependent Γ_s_(*T*), we used the empirical formula obtained by fitting the mean values of Γ_h_ in the uncertainty range shown in Fig. 3c for all cases. Along with the experimental data obtained in this study (circles), the data reported in previous ultrafast TRKR studies on 1L-WSe_2_^38,46^ are also plotted as rectangles and triangles. As shown in Fig. 5, all of the data sets of *τ*_v_ obtained in the experiments conducted under various excitation density conditions could be reproduced with appropriate *N*_x_ and *E*_F_ values as fitting parameters. The *N*_x_ values required to reproduce the data in ref. ^38^ (1.0 × 10^12^ cm^−2^) and ref. ^46^ (2.5 × 10^12^ cm^−2^) are in excellent agreement with the actual excitation densities used in these studies, which are on the orders of ~10^12^ cm^−2^^38^ and ~2 × 10^12^ cm^−2^^46^. The ability to reproduce the experimental data for a wide range of excitation density conditions indicates the robustness and universality of the presented model even under high-exciton-density conditions. Our findings thus offer a unified framework to predict the exciton valley relaxation of 1L-TMDCs observed under various experimental conditions. The results also provide clear guidelines for controlling the excitonic valley relaxation times via Fermi energy (carrier density) tuning and/or linewidth modulations via materials engineering and/or via optical means, which will facilitate the development of opto-valleytronic technologies based on 2D semiconductors.

## Methods

### Sample preparation

We used the mechanical exfoliation method to prepare the 1L-WSe_2_ samples on quartz substrates or on 300-nm-thick SiO_2_/Si substrates^72^. The 1L-WSe_2_ samples stacked on multilayer graphene flakes were fabricated through a dry-transfer method using dimethylpolysiloxane (PDMS) films^73,74^. The 1L-WSe_2_ and multilayer graphene flakes were mechanically exfoliated onto the PDMS films, and these flakes were stacked onto a quartz substrate under an optical microscope.

### Optical measurements

For the circularly polarized excitation of 1L-WSe_2_, white light from a super-continuum light source was sent through a monochromator, a linear polarizing prism, a quarter-wave plate, and a 0.5 numerical aperture objective lens. The *σ*_+_ (*σ*_−_) circularly polarized light was used for the initial excitation of the excitons with the valley pseudospin |+*K*〉 (|−*K*〉). The light emission from the sample was collected using the objective lens, and the *σ*_+_ and *σ*_–_ circularly polarized components were converted into two orthogonal linear polarized components using the quarter-wave plate. These linear polarized components were separated by a calcite polarizing beam displacer and sent to a spectrograph and detected on separated regions of a liquid-nitrogen-cooled charge-coupled device detector. A time-correlated single-photon counting method was used to perform the time-resolved PL spectroscopy under a pulsed excitation of duration of 20 ps and a frequency of 40 MHz. In the time-resolved measurements, the emitted light was sent though band-pass filters (10-nm band width) that corresponded to the exciton resonance energies at each temperature and they were detected using a fiber-coupled single-photon avalanche photodiode device. Except for the results shown in Fig. 2b and Supplementary Fig. 2, light polarization was not resolved in the time-resolved PL measurements. For the temperature range below ~40 K, the fast components of the PL decays shown in Fig. 2c were approximately at the edge of the time resolution limit, whereas the slow components were more than a few hundreds of picoseconds. Thus, the values obtained for 〈*τ*_x_〉 in the low-temperature range may be an upper limit. The excitation power density was 10^2^ W cm^−2^ for both the spectral and time-resolved measurements, except for the excitation-power-dependent measurements shown in Supplementary Fig. 7. We confirmed the linear dependence of the exciton PL intensity on the excitation density. The exciton linewidth exhibited no detectable broadening as the excitation power density increased in the vicinity of the conditions employed in this study (~10^2^ W cm^−2^) (except for the excitation-power-dependent observations using a power density >~4 × 10^2^ W cm^−2^ shown in Supplementary Fig. 7).

### Linewidth analysis

We evaluated the homogeneous linewidths, Γ_h_, of excitons using a fitting procedure with Voigt functions (convolutions of Lorentzian and Gaussian functions). The inset in Fig. 2a and Supplementary Figure 4 show the PL spectra that were decomposed by the Voigt fit at 40 K (Fig. 2a) or at four representative temperatures (Supplementary Fig. 4), respectively. The black circles are the experimental data and the green curves are the spectra reproduced by the peak fit. We considered the peak features for excitons (red curves) and the neighboring peaks of trions^34^ (gray curves) for the spectral decomposition; the major contributions of the neutral exciton (~1.740 eV) and negative trion (~1.706 eV), and one minor peak appearing at about 17 meV below the exciton peak (~1.723 eV) were assumed. The minor PL feature at this energy range was also observed in the carrier-density-dependent PL spectra of 1L-WSe_2_^34,59^; the peak position implies a residual contribution from locally generated negative or positive trions with shifted energies at positions with relatively low carrier density, presumably because of spatial inhomogeneity of the carrier density on the 1L-WSe_2_ on a quartz substrate. Disappearance of this minor component for the high-carrier-doped sample on the atomically flat surface of the multilayer graphene shown in Fig. 4 supports this interpretation. Further details of the fitting procedure are described in Supplementary Note 5.

### Modeling for the temperature dependence of 〈*τ*_x_〉

For convenience, in the discussion and validation of the model diagram shown in Fig. 1b, we considered a phenomenological formula to reproduce the experimental temperature dependence of 〈*τ*_x_〉. Here, we model the temperature dependence of 〈*τ*_x_〉 assuming a finite rate for phonon-mediated exciton scattering between the bright and dark states. We considered processes indicated by arrows in Fig. 1b as the major exciton scattering pathways to express a non-equilibrium bright exciton distribution in a steady-state condition; the expression for 〈*τ*_x_〉 is obtained as:

5 $$\left\langle {\tau _{\mathrm{x}}} \right\rangle \equiv \left[ {\Gamma _{\mathrm{b}} + \frac{{\Gamma _{\mathrm{d}}\exp \left( {\Delta _{{\mathrm{bd}}}/k_{\mathrm{B}}T} \right)}}{{1 + \left( {\Gamma _{\mathrm{d}}/\gamma } \right)\left[ {\exp \left( {\Delta _{{\mathrm{bd}}}/k_{\mathrm{B}}T} \right) - 1} \right]}}} \right]^{ - 1},$$

where Γ_b_ and Γ_d_ are the decay rates of the bright exciton and the dark exciton, respectively (see Supplementary Note 1 for the detailed derivation of this expression), and *γ* is the rate constant for the intervalley phonon scattering. The solid curves in Fig. 3d are the fitted curves reproduced using Eq. (5). The fitted curve excellently reproduced the experimental temperature dependence of 〈*τ*_x_〉 with an assumption of the previously reported bright–dark energy splitting Δ_bd_ in the range from 30 to 47 meV in 1L-WSe_2_^54–57^.

### Data availability

The data that support the findings of this study are available from the corresponding authors on reasonable request.

## Electronic supplementary material

Supplementary Information
