# Supplementary material for: Evidence for line width and carrier screening effects on excitonic valley relaxation in 2D semiconductors
Source: Nat Commun. 2018 Jul 3;9:2598. doi: 10.1038/s41467-018-04988-x (PMC6030139; doi:10.1038/s41467-018-04988-x)
Supplement: Supplementary file 1 — Supplementary Information [file 41467_2018_4988_MOESM1_ESM.pdf]

Supplementary Note 1: Time-integrated valley polarization of excitons

Steady-state exciton valley polarization ρ_x has been deduced in previous literatures[1-3] from standard rate equation models (in which only bright excitons are considered) as,

$$\rho_x = \frac{\rho_0}{1+\tau_x/\tau_v} \quad , \tag{1}$$

where ρ_0, τ_v are the initial valley polarization and the valley relaxation time for the bright excitons, respectively. τ_x is the population lifetime of the bright excitons; γ_r times τ_x corresponds to a PL quantum yield, where γ_r is the radiative decay rate of the bright exciton.

Here, we consider the steady-state exciton valley polarization for the system with multiple exciton levels including bright and dark states as shown in Fig. 1 in the main text. Γ_b and Γ_d are the relaxation rates of the bright and dark excitons to the ground state, respectively. For simplicity, Γ_b and Γ_d are assumed to be independent of temperature. γ is a phonon scattering rate constant, n is a phonon number $n \equiv [\exp(E_{ph}/k_BT) - 1]^{-1}$, where E_{ph} is the phonon energy that corresponds to the energy splitting of the bright and dark states, Δ_{bd}. G_+ and G_- are effective generation rates of bright excitons in $+K$ and $-K$ valleys, respectively, and γ_s is the intervalley scattering rate of bright excitons (holes), considered to be dominated by the electron–hole exchange interactions.

In regards to the possible origin of the lower energy dark states, both intervalley dark excitons ($\mathbf{k} \neq \mathbf{0}$) with electron-spin conservation or intravalley dark excitons ($\mathbf{k} \approx \mathbf{0}$) with spin flip can be considered[4-9]. Here we do not attempt to provide a rigorous discussion as to where the lower energy dark state originates from, but the generation of intervalley dark excitons with spin conservation is expected to be dominant, because of the availability of relevant phonons[5]. Intravalley phonon-assisted scattering with electron spin-flip could also be considered, but this process has been predicted to be mitigated for the monolayer sample supported on a substrate[10]. In Fig. 1, we thus tentatively assumed that the electron-spin-conserving processes are dominant

for the scattering between the bright and dark states. However, we do not rigorously exclude the possibility of the intravalley spin flip processes. For the valley depolarization of holes (excitons), we consider the intervalley scattering of singlet-like bright excitons ($\mathbf{k} \approx \mathbf{0}$) with the valley pseudospins $|-K\rangle$ or $|+K\rangle$ *via* long range electron–hole (*e*–*h*) exchange interactions to be the dominant valley depolarization mechanism at low temperature conditions considered in this study. Although intervalley scattering of holes with spin-flip processes assisted by zone-edge phonons have also been discussed as the possible origin of the exciton valley depolarization[11], such intervalley spin-flip process is forbidden by the selection rules at the lowest-order[10]. Thus, under low temperature conditions focused in this study, contribution of the intervalley scattering of excitons with hole spin-flip due to zone-edge phonon scattering is expected to be smaller than that of the electron-hole exchange interactions that should be effective at the lowest-order, and we neglected this process in our theoretical treatment as a first approximation. Under relatively high temperature conditions ($T > \sim 200$ K), however, multiple intervalley scattering processes through thermal activation[4] or carrier-carrier scattering[2] could also be efficient, and the electron-hole exchange interaction may not be the unique mechanism for the exciton (hole) valley relaxation.

The effects of the exciton scattering between the bright and dark states are taken into account through the consideration of phonon-assisted scattering between the bright and dark states by the use of a finite scattering rate constant, γ. The rate equations corresponding to the scattering processes shown in Fig. 1(b) are below:

$$\frac{dN_{\text{b-}}}{dt} = G_{-} - [\Gamma_{\text{b}} + \gamma(n_{\text{ph}} + 1) + \gamma_{\text{s}}]N_{\text{b-}} + \gamma n_{\text{ph}} N_{\text{d-}} + \gamma_{\text{s}} N_{\text{b+}}, \quad (2)$$

$$\frac{dN_{\text{b+}}}{dt} = G_{+} - [\Gamma_{\text{b}} + \gamma(n_{\text{ph}} + 1) + \gamma_{\text{s}}]N_{\text{b+}} + \gamma n_{\text{ph}} N_{\text{d+}} + \gamma_{\text{s}} N_{\text{b-}}, \quad (3)$$

$$\frac{dN_{\text{d-}}}{dt} = \gamma(n_{\text{ph}} + 1)N_{\text{b-}} - (\Gamma_{\text{d}} + \gamma n_{\text{ph}})N_{\text{d-}}, \quad (4)$$

$$\frac{dN_{\text{d+}}}{dt} = \gamma(n_{\text{ph}} + 1)N_{\text{b+}} - (\Gamma_{\text{d}} + \gamma n_{\text{ph}})N_{\text{d+}}, \quad (5)$$

where $N_{b\pm}$ and $N_{d\pm}$ are the numbers of bright and dark excitons in which holes composing the excitons are at the $+K$ and $-K$ valleys in the electronic Brillouin zone, respectively. The above formulation eventually does not distinguish the spin configuration of the lower lying dark excitons as far as the holes are in the same valley. Therefore, the scattering rate constant γ is defined as an effective quantity, and the analytical result is not affected by whether the scattering processes between the bright exciton and the intravalley dark excitons (not indicated in Fig. 1) are included or not.

Then, under the steady-state condition, we obtain the following two equations:

$$N_{b+} + N_{b-} = (G_+ + G_-)\langle \tau_x \rangle, \tag{6}$$

$$N_{b+} - N_{b-} = \frac{(G_+ - G_-)\langle \tau_x \rangle}{1 + 2\gamma_s \langle \tau_X \rangle}, \tag{7}$$

where we define a quantity, $\langle \tau_x \rangle$, by the following equation:

$$\langle \tau_x \rangle \equiv \left[\Gamma_b + \frac{\Gamma_d \exp(\Delta_{bd} / k_B T)}{1 + (\Gamma_d / \gamma)[\exp(\Delta_{bd} / k_B T) - 1]} \right]^{-1}. \tag{8}$$

Using Supplementary Eqs. (6–8), the valley polarization of the excitons ρ_x can be derived as

$$\rho_x = \frac{N_{b+} - N_{b-}}{N_{b+} + N_{b-}} = \frac{G_+ - G_-}{G_+ + G_-} \frac{1}{1 + 2\gamma_s \langle \tau_x \rangle}. \tag{9}$$

Further defining $\rho_0 \equiv (G_+ - G_-) / (G_+ + G_-)$ and $\tau_v \equiv (2\gamma_s)^{-1}$, the expression of the valley polarization in the main text (Eq. (1)) can be obtained as the following equation:

$$\rho_x = \frac{\rho_0}{1 + \langle \tau_x \rangle / \tau_v}. \tag{10}$$

Supplementary Eq. (6) suggests the physical meaning of the quantity, $\langle \tau_x \rangle$. Modification of Supplementary Eq. (6) gives the following relationship:

$$\langle \tau_x \rangle = \frac{N_{b+} + N_{b-}}{G_+ + G_-} \quad . \tag{11}$$

This expression clearly indicates that $\langle \tau_x \rangle$ is the total number of bright excitons divided by the total exciton generation rate, in other words, the total (integrated) time in which an exciton can be in its bright state before it recombines radiatively or nonradiatively. Thus, γ_r times $\langle \tau_x \rangle$ corresponds to the PL quantum yield of a bright exciton ($\mathbf{k} \approx \mathbf{0}$) generated in the $\pm K$ valley in the electronic Brillouin zone, where γ_r is the radiative decay rate of the bright excitons. In this study, we evaluated this quantity using a photoluminescence (PL) decay profile $I(t)$, because $I(t)$ yields a quantity, as described below:

$$\langle \tau \rangle \equiv \frac{\int_0^\infty I(t)\,dt}{I(0)}, \tag{12}$$

where $I(0)$ is the PL intensity at $t = 0$. The numerator in Supplementary Eq. (12) is proportional to the time-integrated number of the bright excitons, and $I(0)$ is proportional to the total exciton number generated at $t = 0$. As a result, γ_r times $\langle \tau \rangle$ also corresponds to the PL quantum yield of the excitons, and $\langle \tau_x \rangle$ can be evaluated from $\langle \tau \rangle$.

The excellent fit results of the temperature-dependence of $\langle \tau_x \rangle$ using Supplementary Eq. (8) (Eq. (5) in the main text) with Δ_{bd} = 30 meV[5] or 47 meV[12] are shown in Fig. 3(d) in the main text, which supports the validity of the model. Supplementary Eq. (8) suggests that $\langle \tau_x \rangle^{-1}$ at the low-temperature limit corresponds to the sum of γ and Γ_b; namely, the decay rates to the intrinsic dark states (γ) and to extrinsic non-radiative (and intrinsic radiative) channels (Γ_b). The extrinsic decay channels may include the generation of trions and decay into the defect trap states. The fitting shown in Fig. 3(d) also yields the best estimates of the fit parameters as being γ^{-1} ~ 31 ps (35 ps), Γ_b^{-1} ~ 76 ps (68 ps), and Γ_d^{-1} ~ 6.1 ns (75 ns) for Δ_{bd} = 30 meV (47 meV); these values suggest that γ dominates the low-temperature values of $\langle \tau_x \rangle$, and the dark state has a much longer lifetime than that of the bright state.

Here we discuss the limitation of the model. Although the fitting of the experimental values of $\langle \tau_x \rangle$ using Supplementary Eq. (8) (Eq. (5) in the main text) seems to be in successful, it should be noted that the absolute values of the fit parameters may not be so reliable, because there are various neglected factors in the model, e.g., the existence of various lower lying states other than the dark exciton states shown in Fig. 1. In the carrier-doped samples, charged excitons (trions) with the lower energy than the bright excitons can also be generated through the process in which an exciton captures a doped carrier. The trion states could also function as reservoirs of valley-polarized holes similar to the dark exciton states, because the valley relaxation time of trions is predicted to be much longer than that of the bright exciton states[1]. At this stage, since the branching ratio of the bright excitons into the dark or trion states is unknown, further inclusion of various lower lying states into the phenomenological model is not meaningful. Thus, we only assumed the dark exciton states, in which electrons and holes are in the $\pm K$ valleys, as the hole-valley reservoirs in the above model (deep momentum-forbidden dark exciton states[13] in which holes and electrons are at K and Λ valleys, respectively, are also not considered in the model). We also assumed that Γ_b and Γ_d are independent of temperature, although there remains a possibility that these quantities depend on the temperature. These simplifications may considerably affect the obtained values of the fitting parameters. Nevertheless, we also note that the Eq. (1) in the main text (also Supplementary Eq. (10)) is valid as far as the exciton (hole) valley relaxation is dominated by that of the bright excitons.

Finally, we briefly comment on the effect of the existence of the lower lying states for the exciton valley relaxation. The prominent distribution of the excitons in the dark states[5,12,14,15] (or trion states) at low temperatures effectively extends the valley lifetime of holes composing excitons; this has been discussed as being the major origin of the previously reported long persistent component (>150 ps) observed in the time-resolved Kerr rotation signals in 1L-WS_2[14], in which the dark exciton states are expected to lie below the bright states in a manner similar to that found in 1L-WSe_2. In contrast, in 1L-MoS_2 in which the bright–dark

order is predicted to be opposite[7], the net hole valley relaxation time is expected to be comparable to the pure bright exciton valley relaxation time τ_v.

Supplementary Note 2: Excitation photon energy dependence of the valley polarization at 15 K

Supplementary Figure 1 shows the results for the polarization- (Supplementary Fig. 1(a)) and time-resolved (Supplementary Fig. 1(e)) PL measurements under variable excitation photon energies measured for 1L-WSe_2 on a quartz substrate at 15 K. Relatively high valley polarization was observed at the excitation photon energy resonant with the 2s excited exciton energy[16,17] (Supplementary Fig. 1(b)). Under the 2s-resonant excitation condition, slightly narrower line width (Supplementary Fig. 1(c,d)) and shorter $\langle\tau_x\rangle$ (Supplementary Fig. 1(e,f)) than those measured under the non-resonant conditions were observed. The narrower line width may be understood as a consequence of the direct resonant excitation to the 2s exciton band bottom, for which the momentum ($\hbar\mathbf{k}$) distribution of the initially generated 2s excitons could be narrow because only excitons with very small momentum around $\mathbf{k} = 0$ can be excited by the direct optical excitations. Then, if there exist efficient momentum-conserving decay pathways from the 2s to 1s excitons, narrow momentum distribution (thus narrow line width) in the 1s exciton band may also be achieved. However, detailed mechanisms for these excitation energy dependent variations are still unclear and remain to be addressed.

Using the obtained Γ_h and $\langle\tau_x\rangle$ at each excitation photon energy as inputs to Eq. (4) in the main text, relatively large valley polarization ρ_x near the 2s-resonant excitation conditions could be reproduced (Supplementary Fig. 1(g)). The mean value of ρ_x in the prediction band (orange shaded region) seems to be larger than the experimental values for the excitation photon energy of more than ~1.9 eV. Although we have set the initial valley polarization as $\rho_0 = 0.7$ (See Supplementary Note 3) to calculate the prediction band of ρ_x, for the excitation photon

energies of more than ~1.9 eV, the better matching of the prediction band to the experimental data could be obtained by setting ρ_0 = 0.6 (not shown). This implies that the value of ρ_0 in 1L-WSe_2 gradually decreases as the excitation photon energy increases, presumably because the initial valley depolarization potentially caused by the defect-induced intervalley generation[18], ultrafast valley relaxation of hot excitons with a large exciton momentum during their intraband cooling processes[19], and/or excitation to other valleys[20] through phonon-assisted indirect absorption processes[10] within the initial ultrafast time scale, become efficient owing to the large excess energy.

We note that, in the case of the resonant excitation condition of the 1s bright excitons, ρ_0 could be close to 1, and the valley relaxation time could be much longer than that for the non-resonant excitation conditions because of the very narrow momentum distribution of the resonantly photogenerated 1s excitons around **k** = 0. Since this condition is not taken into account, the model is applicable only for the non-resonant excitation conditions.

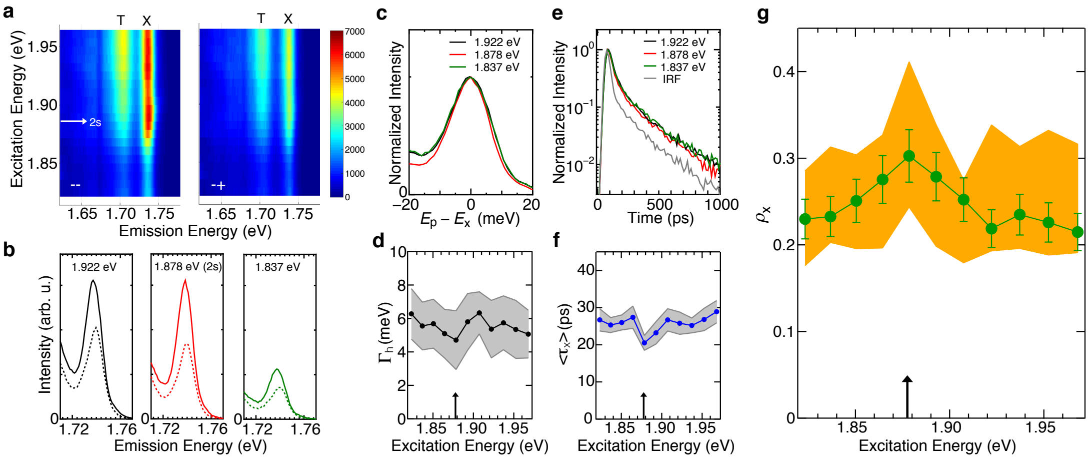

Supplementary Figure 1 | Polarization resolved PL excitation spectroscopy. (**a**) Polarization resolved PL intensities as functions of excitation and emission photon energies measured for a 1L-WSe_2 sample on a quartz substrate at 15 K. X and T indicate exciton and trion PL peaks. The left (−−) and right (−+) panels show σ- and σ+ emission intensities under σ- excitation conditions, respectively. (**b**) Comparison of the polarization resolved exciton PL spectra at various excitation photon energies. Solid

and dotted curves correspond to the same and opposite circular polarization conditions for excitation and detection. (**c**) Comparison of the exciton PL line shapes at typical excitation photon energies. E_p and E_x are photon energy and exciton energy, respectively. (**d**) Γ_h as a function of excitation photon energy. Gray shaded region indicates the reasonable uncertainty range for the Γ_h deduced by the Voigt fitting procedure. (**e**) Time resolved PL decay profiles for the typical excitation photon energies. (**f**) $\langle \tau_x \rangle$ values deduced from the data in (**e**) plotted as a function of excitation photon energy. (**g**) Exciton valley polarization ρ_x obtained from the data shown in (**a**) plotted as a function of excitation photon energy. The orange shaded region is the prediction band calculated using Eq. (4) where Γ_h in (**d**) and $\langle \tau_x \rangle$ in (**f**) were used as input parameters. The error bars correspond to the uncertainties of the polarization resolved measurements. The arrows in (**a**), (**d**), (**f**), (**g**) indicate the excitation photon energy corresponds to 2s exciton resonance.

Supplementary Note 3: Evaluation of ρ_0 from time-dependent polarized PL measurements

Supplementary Figure 2 compares the polarization-resolved PL decay profiles [$I_{\sigma+}$ (red curve) and $I_{\sigma-}$ (black curve)] and the time-resolved valley polarization, $\rho_x(t)$, (green circles) measured at (a) 45 K (same as shown in Fig. 2(b) in the main text) and (b) 120 K under the σ_+ excitation condition for 1L-WSe_2 on a quartz substrate. By extrapolating $\rho_x(t)$ to the onset time ($t \approx 0$ ps) of the PL decay signals (dotted line), a lower limit of the maximum valley polarization $\rho_x(0) \approx 0.7$ was deduced, regardless of the temperature. The persistent valley polarization $\rho_x(t)$ at times longer than a few tens of picoseconds is mainly owing to the slow instrumental response. According to this observation, we set $\rho_0 = 0.7$ for 1L-WSe_2 in all of the analyses using the ρ_0 value. Thus, the potential uncertainty in the estimation of ρ_0 (± 0.1) could cause a finite modification of all the results obtained from the analyses using ρ_0 (absolute values of τ_v in Fig. 3(e) will be changed ± 20-30%), but these modifications do not change the discussions on the underlying physics (See also Supplementary Note 4).

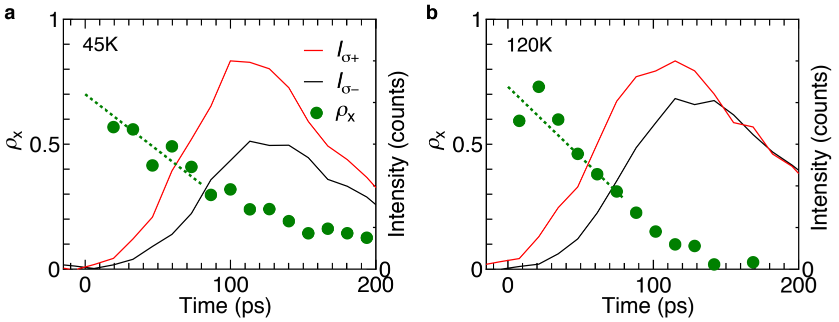

Supplementary Figure 2 | Circular-polarization-resolved PL decay profiles. (**a**,**b**) Data measured at (**a**) 45 K and (**b**) 120 K for 1L-WSe_2 on a quartz substrate. The excitation photon energy was 1.823 eV. The time-resolved valley polarization, $\rho(t)$, was obtained from the PL decay profiles of $I_{\sigma+}(t)$ and $I_{\sigma-}(t)$ as $\rho(t) = [I_{\sigma+}(t) - I_{\sigma-}(t)] / [I_{\sigma+}(t) + I_{\sigma-}(t)]$. The dotted lines are extrapolations of $\rho(t)$ to $t \approx 0$ ps. A 1L-WSe_2 on a quartz substrate of a different sample batch from that in Fig. 2(a) and Fig. 2(c) in the main text was used for these measurements.

Supplementary Note 4: Effects of the uncertainty in ρ_0

The potential uncertainty in the estimation of ρ_0 (0.7 ± 0.1) could cause modifications of the results obtained from the analyses using the ρ_0. Thus, here we discuss the effects of the different ρ_0 values on the analysis in this study. Supplementary Figure 3(a) shows the temperature-dependent valley relaxation times τ_v obtained using Eq. (1) in the main text for ρ_0 = 0.6, 0.7 and 0.8, respectively. The ρ_0 value of 0.6 (0.8) yields τ_v of ~13.5 (~8.5) ps at low temperature. These changes also modify the J parameter for the exchange interaction strength; for τ_v obtained using $\rho_0 = 0.6$ (0.8), $J = 0.74$ (0.91) eV in Eq. (2) in the main text is required to reproduce the experimental results. The above modification also results in the change of the parameter C in Eqs. (3) and (4) in the main text; for $\rho_0 = 0.6$ (0.8), $C \approx 152$ (96) is obtained. The parameter E_F is also modified. Supplementary Figure 3(b) shows the temperature-dependent valley relaxation times $\tau_v(T)$ obtained for the ρ_0 in the range of 0.6-0.8, normalized by the low temperature values at 10K (τ_v(10K)). The E_F values in the range of 12-15 meV, which

correspond to the carrier density in the range of about 1.9-2.4 × 10^{12} cm^{-2}, can reproduce the experimental results (solid curves in Supplementary Figure 3(b)). The J and E_F parameters obtained for the different ρ_0 values (0.7 ± 0.1) are still in the reasonable range. Thus, these modifications caused by the uncertainty in the ρ_0 parameter do not change the discussions on the underlying physics.

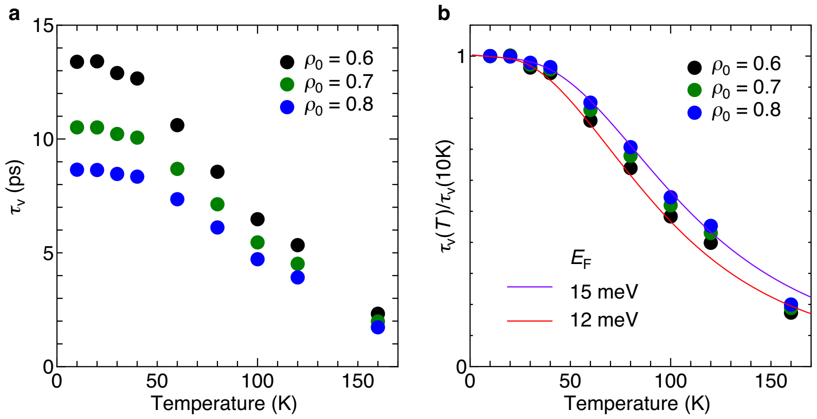

Supplementary Figure 3 | Exciton valley relaxation times τ_v deduced using various ρ_0. (**a**) Exciton valley relaxation times τ_v deduced for the ρ_0 of 0.6 (black circles), 0.7 (green circles), and 0.8 (blue circles) using Eq. (1) in the main text plotted as functions of temperature. (**b**) The τ_v normalized by their values at 10 K, τ_v(10K), plotted as functions of temperature. The solid curves in (**b**) are reproduced using Eq. (3) in the main text. For the temperature dependent Γ_h in Eq. (3), the empirical fit function plotted in Fig. 3(c) in the main text was used for clarity.

Supplementary Note 5: Line width analysis

We evaluate the homogeneous line widths, Γ_h, of excitons using a fitting procedure with Voigt functions (which are a convolution of Lorentzian and Gaussian functions). Supplementary Figure 4 shows the PL spectra that were decomposed by the Voigt fit at four representative temperatures; 10 K, 40 K, 80 K, and 160 K. The black circles are the experimental data and the green curves are the data reproduced by the peak fit. We considered the peak features for

excitons (red curves) and the neighboring peaks of various types of trions[21] (gray curves) for the spectral decomposition; the major contributions of the neutral exciton (~1.740 eV) and negative trion (~1.706 eV), and one minor peak appearing at about 17 meV below the exciton peak (~1.723 eV) were considered. The minor PL feature at this energy range was also observed in the carrier density dependent PL spectra of 1L-WSe_2[21,22]. The origin of this PL feature is unclear, but the peak position implies a residual contribution from locally generated negative or positive trions with shifted energies at positions with relatively low carrier density, presumably because of spatial inhomogeneity of the carrier density on the 1L-WSe_2 on a quartz substrate. The energy separations between these peaks were kept unchanged throughout the fit analysis for all of the temperatures. These constraints yielded reasonable fits, which are shown in Supplementary Fig. 4 and inset of Fig. 2(a). In the Voigt fit procedure, we consider the Lorentzian component to be a homogeneous line width, while the Gaussian component reflects inhomogeneous line widths owing to possible inhomogeneity (in local doping level, defect density, etc.) of the material within a focal spot and the finite spectral resolution of our detection setup (3.6 meV for the spectra in Fig. 2 and Fig. 4 in the main text and in Supplementary Fig. 1, and 2.0 meV for the spectra in Supplementary Fig. 7). The temperature-independent Gaussian width in the range of ~11–14 meV was found to yield reasonable fits for the data shown in Fig. 2 at all of the temperatures between 10–160 K, as shown in Supplementary Fig. 4. The Lorentzian widths determined for the lower (upper) limit of the constant Gaussian width of 10.8 meV (14.3 meV) were considered to be the upper (lower) limit of the Lorentzian widths at each temperature in the analyses. Similar procedure was also used for the line width analysis on the data shown in Fig. 4 (Supplementary Fig. 7), for which the constant Gaussian width of ~10–12 meV (~9 meV) was found to yield reasonable fit results.

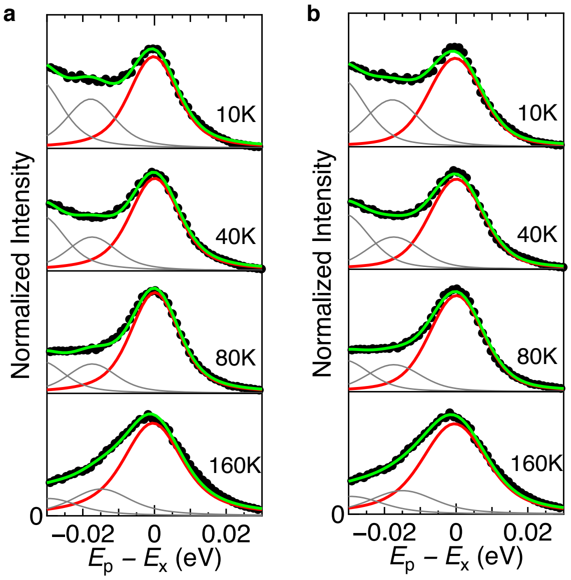

Supplementary Figure 4 | Results of the Voigt fit. (**a**,**b**) Results for constant Gaussian widths of (**a**) 10.8 meV and (**b**) 14.3 meV. The black circles represent the experimental data at each temperature, while the red, gray, and green curves correspond to the exciton peaks, trion peaks, and the sum of all of the model peaks, respectively. All of the spectra are plotted as functions of the difference of the photon energy (E_p) and exciton peak energy (E_x) for comparison.

Supplementary Note 6: PL decay profiles in linear plots

Supplementary Figure 5 shows the PL decay profiles at 10K, 40K, 80K, and 160K in linear plots.

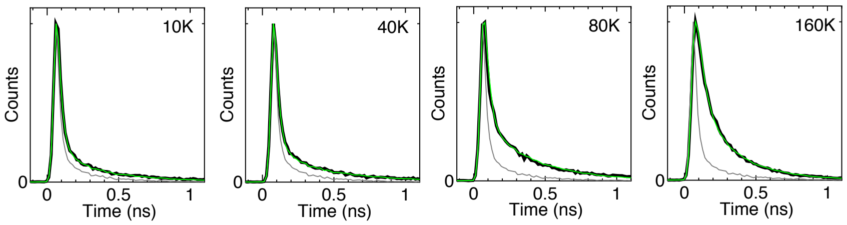

Supplementary Figure 5 | PL decay profiles in linear plots. Typical experimental PL decay profiles (black curves) fitted with the convolution of the double exponential model decay function and instrumental response function (IRF, gray curves). The calculated

decay profiles for four representative temperatures (10 K, 40 K, 80 K and 160 K) are shown as light green curves.

Supplementary Note 7: Comparison of the temperature dependences of the $\langle\tau_x\rangle$ and the exciton PL intensity

Here we compare the temperature dependences of the $\langle\tau_x\rangle$ and the integrated intensity of bright exciton emission I_x at low temperature conditions (Supplementary Figure 6) to confirm that these quantities show the expected relation of $I_x \propto \gamma_r\langle\tau_x\rangle$, where γ_r is the radiative decay rate. Under the conditions in which $\Gamma_h > k_BT$ is fulfilled, γ_r in two-dimensional systems has been predicted to follow $\gamma_r \propto [1 - \exp(-\Gamma_h/k_BT)] / \Gamma_h \equiv r(T)$[23,24]. Thus, $I_x \propto r(T)\langle\tau_x\rangle$ is expected. This expression was derived by considering the effects of intraband thermalization of excitons, exciton coherence length limited by collisions (that also yield Γ_h), and the corresponding uncertainty in the exciton momentum that determines the range of exciton states that can contribute to the optical transitions[23,24]. We note that it becomes $r(T) \propto 1/T$ in the coherent limit, $\Gamma_h << k_BT$, which is commonly fulfilled in the case of conventional semiconductor quantum wells (in the limit of $\Gamma_h \rightarrow 0$, exciton states only within the light cone can contribute to the optical transitions). As expected, the temperature dependent variation of the I_x at low temperatures is in good agreement with that of $r(T)\langle\tau_x\rangle$; this confirms the consistency between the quantities $\langle\tau_x\rangle$ and I_x obtained in the experiments.

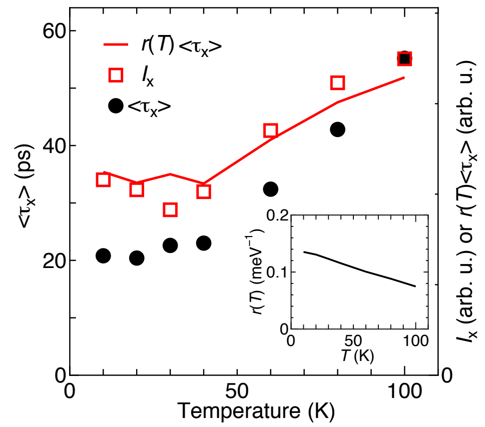

Supplementary Figure 6 | $\langle\tau_x\rangle$, I_x, and $r(T)\langle\tau_x\rangle$ plotted as functions of temperature. Inset shows the $r(T)$ as a function of temperature. The mean values of Γ_h in the reasonable range shown in Fig. 3(c) in the main text were used to calculate $r(T)$.

Supplementary Note 8: τ_v under the condition without carrier screening effect

Here we evaluate the τ_v for 1L-WSe_2 under the condition with no carrier screening[25]; τ_v determined by the unscreened *e-h* exchange interaction strength in the range of $J \sim 0.5$ - 1 eV is predicted to be $\tau_v = M/A\hbar J^2 \sim 2.5$ - 10 fs and independent of the temperature[25]. Thus, τ_v determined by the unscreened *e-h* exchange interaction is much shorter than the value calculated with the carrier screening effect, and unrealistic considering the observable valley polarization of the excitons for $\langle\tau_x\rangle$ more than 10 ps ($\tau_v \sim 10$ fs and $\langle\tau_x\rangle \sim 10$ ps result in negligible valley polarization $\rho_x \sim 10^{-3}$ according to Eq. (1)), which clearly contradict the experimental observations.

Supplementary Note 9: Evaluation of the naturally doped carrier density

The emergence of the negative trion peaks in the PL spectra shown in Fig. 1 indicates that 1L-WSe_2 was in a naturally electron-doped condition as discussed in the main text. Here we provide an estimation of the naturally doped electron density from the ratio of the observed trion

and exciton PL intensities at 160 K at which thermal equilibrium condition is approximately fulfilled. According to the mass action law[26-28], electron density, n_c, is related to the exciton (N_x) and trion (N_{x-}) populations in the equilibrium conditions as

$$\frac{N_x n_c}{N_{x-}} = \frac{4M_x m_e k_B T}{\pi \hbar^2 M_{x-}} \exp\left(-\frac{E_{bx-}}{k_B T}\right) , \tag{13}$$

where m_e, M_x (~$2m_e$), M_{x-} (~$3m_e$) are electron, exciton, and trion masses, respectively, and E_{bx-} is the trion binding energy. The relation between the exciton (I_x) and trion (I_{x-}) intensities and their populations N_x and N_{x-} are expressed as

$$\frac{I_{x-}}{I_x} = \frac{\gamma_{x-} N_{x-}}{\gamma_x N_x} , \tag{14}$$

where γ_x and γ_{x-} are the effective radiate decay rate of excitons and trions, respectively. Using Supplementary Eqs. (13) and (14), n_c is evaluated as

$$n_c = \left(\frac{\gamma_x}{\gamma_{x-}}\right) \cdot \left(\frac{I_{x-}}{I_x}\right) \cdot \left(\frac{4M_x m_e k_B T}{\pi \hbar^2 M_{x-}}\right) \exp\left(-\frac{E_{bx-}}{k_B T}\right), \tag{15}$$

Using Supplementary Eq. (15) with the experimental values of (I_{x-}/ I_x) ~ 0.2 and E_{bx-} ~ 30 meV at T = 160 K, n_c ~ (γ_x/γ_{x-}) × 10^{11} cm^{-2} in 1L-WSe_2 is obtained. The ratio of the effective radiative decay rates of excitons and trions, γ_x/γ_{x-}, has been evaluated to be on the order of 1-10 for the related material 1L-MoS_2 [27-29]. Thus, we estimate that the lower bound of the naturally doped electron density is on the order of n_c ~ 10^{11} - 10^{12} cm^{-2} at 160 K; this is consistent with the electron density obtained by the fit using Eq. (2) in the main text, and supports the validity of taking the carrier screening effects into account for the evaluation of exciton valley lifetimes in naturally doped 1L-WSe_2 rather than assuming non-doped conditions[30].

Supplementary Note 10: Material, doping, and substrate dependences of the C coefficient

Since $C \equiv k_{\mathrm{TF0}}^2/\mathrm{A}J^2 \approx g_s^2 g_v^2 e^4/144a^2\varepsilon^2 J^2$, the constant C in Eq. (3) and Eq. (4) does not explicitly depend on the exciton (or carrier) effective mass, and should scale as $C \propto a^{-2}\varepsilon^{-2}J^{-2}$. Considering $J \approx 8\pi^2 aE_b t^2/3a_B E_g^2$, the approximate scaling of C is predicted as

$$C \propto \left(\frac{a_{\mathrm{B}}}{a}\right)^4 \left(\frac{E_{\mathrm{g}}}{t}\right)^4 . \tag{16}$$

Since the differences in lattice constants a (~0.32-0.34 nm) for typical 1L-TMDCs (e.g. MoS_2, $MoSe_2$, WS_2, WSe_2) are small, the exciton Bohr radius a_B (or dielectric constant ε and exciton binding energy E_b via the approximate relation $E_b \propto (\varepsilon a_B)^{-1}$), and the ratio of the band gap and hopping integral (E_g/t) are inferred as the critical factors to determine C for each material. $(E_g/t)^4$ can be estimated as about 5.19, 5.98, 2.91 and 3.27 for MoS_2, $MoSe_2$, WS_2 and WSe_2, respectively, using the results from first-principles band structure calculations[31]. Thus, if a_B for these materials are comparable, the C values for MoX_2 could be about twice as large as those for WX_2 (X = S or Se). However, the above simple prediction does not explain the previously reported vanishing valley polarization in PL for $MoSe_2$[6,32,33], in which additional factors, such as polaron-induced Rabi oscillations between bright and dark excitons[6], should also be considered to explain the anomalous exciton valley relaxation phenomena.

The C parameter may also depend on the carrier density. Since Supplementary Eq. (16) suggests that the constant C scales as $C \propto a_B^4 E_g^4$, the possible changes in a_B ($\propto \varepsilon^{-1}E_b^{-1}$) and E_g under the carrier doping condition determines how the C parameter varies depending on the carrier density. According to the previous studies[34,35], the exciton binding energy E_b and the quasi-particle gap E_g vary depending on the carrier density owing to the carrier screening considerably weakened by the dynamical effect[35]. It is noted that the dynamical screening effect has been predicted to be important on the direct term of the Coulomb interaction[35,36] that mainly determines the exciton energy, whereas its impact on the e–h exchange interaction that is

responsible for the exciton valley relaxation in the current study still remains an open question. For the change of the carrier density from ~2 × 10^{12} to ~3 × 10^{12} cm^{-2}, reduction of E_b and E_g on the order of 1 % and 0.1 %, were observed[34], respectively. It is roughly estimated that the 1% reduction of the E_b leads to the reduction of the width of the exciton wave function in the k space on the order of 0.5% for the parabolic dispersion relation (because $\Delta k/k \approx (1/2)\Delta E/E$ for small $\Delta E/E$). This may cause about 0.5% increase of the a_B through the uncertainty relation. Thus, the change in the C parameter by the carrier density increase from ~2 × 10^{12} to ~3 × 10^{12} cm^{-2} is roughly estimated to be by a factor of $(1.005)^4(0.999)^4 \approx 1.02$, namely, about 2% increase of the C parameter is expected.

The additional screening in a realistic dielectric environment (such as the existence of quartz or few layer graphene substrates) may also affect the results. Previous theoretical and experimental studies[37] reported that the existence of a thin multilayer graphene substrate reduces about 20% of the exciton binding energy (E_b) and 5% of the quasi-particle band gap (E_g) from their intrinsic values in a free standing 1L-TMDC. It is roughly estimated that the 20% reduction of the E_b leads to the reduction of the width of the exciton wave function in the k space on the order of 10% for the parabolic dispersion relation. This may cause about 10% increase of the a_B through the uncertainty relation. Thus, the C parameter of the sample on graphene is expected to be larger than that of the intrinsic free standing 1L-TMDCs, roughly by a factor $(1.1)^4(0.95)^4 \approx 1.19$, namely, enhancement of about 20%. Because of the metallic screening in the graphene, the effective dielectric constant for the graphene substrate is expected to be larger than that for the quartz substrate. Based on the above consideration, we estimate that the variation of the valley relaxation time for the different substrates (quartz or graphene) is at most within 20 %, and should be smaller than 20% because the dielectric screening by the quartz substrate (with bulk dielectric constant of ~ 4) should also be larger than that of vacuum; thus the difference between the graphene and the quartz substrates should be smaller than that between the graphene and vacuum.

Supplementary Note 11: Linewidth dependence of the exciton valley polarization

In the discussions of the main text, we demonstrated that our framework based on the intervalley scattering of the bright excitons via the screened *e–h* exchange interaction can excellently reproduce the temperature-dependence of the experimental valley polarization and τ_v including the saturation plateau behavior under low temperature conditions. However, phonon-assisted (thermally-activated) intervalley scattering mechanism[4,11], that may be gradually important as the temperature increased, might also affect the temperature-dependent exciton valley relaxation. To further confirm the dominance of the screened *e–h* exchange mechanism at low temperature, we examined the exciton linewidth dependence of the valley polarization at a nearly constant temperature condition by changing the excitation power densities. This experiment enables to directly evaluate the linewidth dependence of the valley polarization without changing the phonon number. Supplementary Fig. 7(a) shows the PL spectra of 1L-WSe_2 exfoliated on SiO_2/Si substrate measured under relatively low (0.4 kW cm^{-2}) and high (2.5 kW cm^{-2}) excitation power densities at 10 K. This sample showed a narrower line width than that of 1L-WSe_2 shown in Fig. 2 and the exciton peak was well separated from other PL features; this allowed us to evaluate the small change in the exciton line width with better accuracy. As shown in Supplementary Fig. 7(a), the exciton peak position was almost unchanged within the power density range examined in this study; this suggests that the sample temperature was not increased even under the highest excitation power density employed in the measurements. Unfortunately, the effective lifetime $\langle \tau_x \rangle$ for this sample could not be obtained because it was faster than the detection limit. Instead, we observed excitation power dependence of the integrated PL intensity as shown in Supplementary Fig. 7(b). The integrated intensity showed linear increase as the excitation power increased. This suggests that $\langle \tau_x \rangle$ was almost constant regardless of the excitation power.

Supplementary Fig. 7(c) compares the PL line shapes at low (0.4 kW cm^{-2}) and high (2.5 kW cm^{-2}) power excitation conditions. The spectrum under the high density condition

clearly exhibited line width broadening. Supplementary Fig. 7(d) plots the ρ_x and Γ_h as functions of the excitation power density. Clear anti-correlation between these quantities was observed. Γ_h was increased as the power density increased, while ρ_x decreased. Using Eq. (4) at the low temperature limit with the coefficients ρ_0 = 0.7 and C = 118 ($\approx\tau_v(0)\Gamma_h(0)/\hbar$), the observed reduction in ρ_x could be excellently reproduced from the power density dependent variation in Γ_h with $\langle\tau_x\rangle \approx$ 10 ps as a fit parameter. Thus, within the excitation densities tested in this study, the assumption of the constant ρ_0 regardless of the excitation density seems to be consistent. However, we note that there still remains a possibility that the ρ_0 depends on the exciton density when it is much higher than those examined in this study.

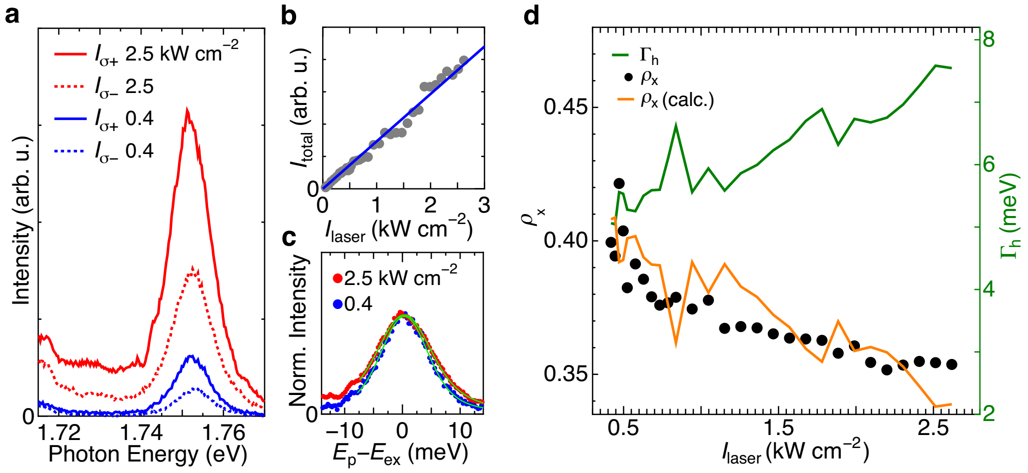

Supplementary Figure 7 | Excitation power dependence in the PL line width and valley polarization in 1L-WSe$_2$ at 10 K. (**a**) Polarization resolved PL spectra of 1L-WSe$_2$ exfoliated on SiO$_2$/Si substrate measured under excitation power densities of 0.4 kW cm^{-2} and 2.5 kW cm^{-2} at 10 K. Excitation photon energy was 1.879 eV. (**b**) Total PL intensities (I_{total} = $I_{\sigma+}$ + $I_{\sigma-}$) as a function of excitation power density $I_{laser.}$ Solid line is a linear fit. (**c**) Comparison of the PL line shape at 2.5 kW cm^{-2} and 0.4 kW cm^{-2}. Solid curves are the results of the Voigt fitting. (**d**) Γ_h (green curve), ρ_x (black solid circles),

and calculated ρ_x (orange curve) using Eq. (4) (at the low temperature limit) in the main text.

Supplementary References

1. Mak, K. F., He, K., Shan, J. & Heinz, T. F. Control of valley polarization in monolayer MoS_2 by optical helicity. *Nat. Nanotechnol.* **7**, 494-498 (2012).
2. Kioseoglou, G., Hanbicki, A. T., Currie, M., Friedman, A. L., Gunlycke, D. & Jonker, B. T. Valley polarization and intervalley scattering in monolayer MoS_2. *Appl. Phys. Lett.* **101**, 221907 (2012).
3. Neumann, A., Lindlau, J., Colombier, L., Nutz, M., Najmaei, S., Lou, J., Mohite, A. D., Yamaguchi, H. & Högele, A. Opto-valleytronic imaging of atomically thin semiconductors. *Nat. Nanotechnol.* **12**, 329-334 (2017).
4. Mai, C., Semenov, Y. G., Barrette, A., Yu, Y., Jin, Z., Cao, L., Kim, K. W. & Gundogdu, K. Exciton valley relaxation in a single layer of WS_2 measured by ultrafast spectroscopy. *Phys. Rev. B* **90**, 041414 (2014).
5. Zhang, X.-X., You, Y., Zhao, S. Y. F. & Heinz, T. F. Experimental evidence for dark excitons in monolayer WSe_2. *Phys. Rev. Lett.* **115**, 257403 (2015).
6. Dery, H. & Song, Y. Polarization analysis of excitons in monolayer and bilayer transition-metal dichalcogenides. *Phys. Rev. B* **92**, 125431 (2015).
7. Echeverry, J. P., Urbaszek, B., Amand, T., Marie, X. & Gerber, I. C. Splitting between bright and dark excitons in transition metal dichalcogenide monolayers. *Phys. Rev. B* **93**, 121107 (2016).
8. Yu, H., Cui, X., Xu, X. & Yao, W. Valley excitons in two-dimensional semiconductors. *Natl. Sci. Rev.* **2**, 57-70 (2015).
9. Palummo, M., Bernardi, M. & Grossman, J. C. Exciton radiative lifetimes in two-dimensional transition metal dichalcogenides. *Nano Lett.* **15**, 2794-2800 (2015).
10. Song, Y. & Dery, H. Transport theory of monolayer transition-metal dichalcogenides through symmetry. *Phys. Rev. Lett.* **111**, 026601 (2013).
11. Zeng, H., Dai, J., Yao, W., Xiao, D. & Cui, X. Valley polarization in MoS_2 monolayers by optical pumping. *Nat. Nanotechnol.* **7**, 490-493 (2012).
12. Zhang, X.-X., Cao, T., Lu, Z., Lin, Y.-C., Zhang, F., Wang, Y., Li, Z., Hone, J. C., Robinson, J. A., Smirnov, D., Louie, S. G. & Heinz, T. F. Magnetic brightening and control of dark excitons in monolayer WSe_2. *Nat. Nanotechnol.* **12**, 883-888 (2017).
13. Selig, M., Berghäuser, G., Raja, A., Nagler, P., Schüller, C., Heinz, T. F., Korn, T., Chernikov, A., Malic, E. & Knorr, A. Excitonic linewidth and coherence lifetime in monolayer transition metal dichalcogenides. *Nat. Commun.* **7**, 13279 (2016).
14. Plechinger, G., Nagler, P., Arora, A., Schmidt, R., Chernikov, A., del Águila, A. G., Christianen, P. C. M., Bratschitsch, R., Schüller, C. & Korn, T. Trion fine structure and coupled spin–valley dynamics in monolayer tungsten disulfide. *Nat. Commun.* **7**, 12715 (2016).
15. Zhou, Y., Scuri, G., Wild, D. S., High, A. A., Dibos, A., Jauregui, L. A., Shu, C., De Greve, K., Pistunova, K., Joe, A. Y., Taniguchi, T., Watanabe, K., Kim, P., Lukin, M. D. & Park, H. Probing dark excitons in atomically thin semiconductors via near-field coupling to surface plasmon polaritons. *Nat. Nanotechnol.* **12**, 856-860 (2017).
16. He, K., Kumar, N., Zhao, L., Wang, Z., Mak, K. F., Zhao, H. & Shan, J. Tightly bound excitons in monolayer WSe_2. *Phys. Rev. Lett.* **113**, 026803 (2014).
17. Chernikov, A., Berkelbach, T. C., Hill, H. M., Rigosi, A., Li, Y., Aslan, O. B., Reichman, D. R., Hybertsen, M. S. & Heinz, T. F. Exciton binding energy and nonhydrogenic Rydberg series in monolayer WS_2. *Phys. Rev. Lett.* **113**, 076802 (2014).
18. Wang, Q., Ge, S., Li, X., Qiu, J., Ji, Y., Feng, J. & Sun, D. Valley carrier dynamics in monolayer molybdenum disulfide from helicity-resolved ultrafast pump–probe spectroscopy. *ACS Nano* **7**, 11087-11093 (2013).
19. Yu, T. & Wu, M. W. Valley depolarization due to intervalley and intravalley electron-hole

exchange interactions in monolayer MoS_2. *Phys. Rev. B* **89**, 205303 (2014).
20. Kozawa, D., Kumar, R., Carvalho, A., Kumar Amara, K., Zhao, W., Wang, S., Toh, M., Ribeiro, R. M., Castro Neto, A. H., Matsuda, K. & Eda, G. Photocarrier relaxation pathway in two-dimensional semiconducting transition metal dichalcogenides. *Nat. Commun.* **5**, 4543 (2014).
21. Jones, A. M., Yu, H., Ghimire, N. J., Wu, S., Aivazian, G., Ross, J. S., Zhao, B., Yan, J., Mandrus, D. G., Xiao, D., Yao, W. & Xu, X. Optical generation of excitonic valley coherence in monolayer WSe_2. *Nat. Nanotechnol.* **8**, 634-638 (2013).
22. Wang, Z., Shan, J. & Mak, K. F. Valley- and spin-polarized landau levels in monolayer WSe_2. *Nat. Nanotechnol.* **12**, 144-149 (2016).
23. Feldmann, J., Peter, G., ouml, bel, E. O., Dawson, P., Moore, K., Foxon, C. & Elliott, R. J. Linewidth dependence of radiative exciton lifetimes in quantum wells. *Phys. Rev. Lett.* **59**, 2337 (1987).
24. Jin, C., Kim, J., Wu, K., Chen, B., Barnard, E. S., Suh, J., Shi, Z., Drapcho, S. G., Wu, J., Schuck, P. J., Tongay, S. & Wang, F. On optical dipole moment and radiative recombination lifetime of excitons in WSe_2. *Adv. Funct. Mater.* **27**, 1601741 (2017).
25. Konabe, S. Screening effects due to carrier doping on valley relaxation in transition metal dichalcogenide monolayers. *Appl. Phys. Lett.* **109**, 073104 (2016).
26. Ross, J. S., Wu, S., Yu, H., Ghimire, N. J., Jones, A. M., Aivazian, G., Yan, J., Mandrus, D. G., Xiao, D., Yao, W. & Xu, X. Electrical control of neutral and charged excitons in a monolayer semiconductor. *Nat. Commun.* **4**, 1474 (2013).
27. Mouri, S., Miyauchi, Y. & Matsuda, K. Tunable photoluminescence of monolayer MoS_2 via chemical doping. *Nano Lett.* **13**, 5944-5948 (2013).
28. Lin, Y., Ling, X., Yu, L., Huang, S., Hsu, A. L., Lee, Y.-H., Kong, J., Dresselhaus, M. S. & Palacios, T. Dielectric screening of excitons and trions in single-layer MoS_2. *Nano Lett.* **14**, 5569-5576 (2014).
29. Wang, H., Zhang, C., Chan, W., Manolatou, C., Tiwari, S. & Rana, F. Radiative lifetimes of excitons and trions in monolayers of the metal dichalcogenide MoS_2. *Phys. Rev. B* **93**, 045407 (2016).
30. Zhu, C. R., Zhang, K., Glazov, M., Urbaszek, B., Amand, T., Ji, Z. W., Liu, B. L. & Marie, X. Exciton valley dynamics probed by Kerr rotation in WSe_2 monolayers. *Phys. Rev. B* **90**, 161302 (2014).
31. Xiao, D., Liu, G.-B., Feng, W., Xu, X. & Yao, W. Coupled spin and valley physics in monolayers of MoS_2 and other group-VI dichalcogenides. *Phys. Rev. Lett.* **108**, 196802 (2012).
32. Wang, G., Palleau, E., Amand, T., Tongay, S., Marie, X. & Urbaszek, B. Polarization and time-resolved photoluminescence spectroscopy of excitons in $MoSe_2$ monolayers. *Appl. Phys. Lett.* **106**, 112101 (2015).
33. MacNeill, D., Heikes, C., Mak, K. F., Anderson, Z., Kormányos, A., Zólyomi, V., Park, J. & Ralph, D. C. Breaking of valley degeneracy by magnetic field in monolayer $MoSe_2$. *Phys. Rev. Lett.* **114**, 037401 (2015).
34. Chernikov, A., van der Zande, A. M., Hill, H. M., Rigosi, A. F., Velauthapillai, A., Hone, J. & Heinz, T. F. Electrical tuning of exciton binding energies in monolayer WS_2. *Phys. Rev. Lett.* **115**, 126802 (2015).
35. Gao, S., Liang, Y., Spataru, C. D. & Yang, L. Dynamical excitonic effects in doped two-dimensional semiconductors. *Nano Lett.* **16**, 5568-5573 (2016).
36. Strinati, G. Effects of dynamical screening on resonances at inner-shell thresholds in semiconductors. *Phys. Rev. B* **29**, 5718-5726 (1984).
37. Ugeda, M. M., Bradley, A. J., Shi, S.-F., da Jornada, F. H., Zhang, Y., Qiu, D. Y., Ruan, W., Mo, S.-K., Hussain, Z., Shen, Z.-X., Wang, F., Louie, S. G. & Crommie, M. F. Giant bandgap renormalization and excitonic effects in a monolayer transition metal dichalcogenide semiconductor. *Nat. Mater.* **13**, 1091-1095 (2014).
